# Transcriptional activation of Jun and Fos members of the AP‐1 complex is a conserved signature of immune aging that contributes to inflammaging

**DOI:** 10.1111/acel.13792

**Published:** 2023-02-24

**Authors:** Emin Onur Karakaslar, Neerja Katiyar, Muneer Hasham, Ahrim Youn, Siddhartha Sharma, Cheng‐han Chung, Radu Marches, Ron Korstanje, Jacques Banchereau, Duygu Ucar

**Affiliations:** ^1^ The Jackson Laboratory for Genomic Medicine Farmington Connecticut USA; ^2^ Leiden University Medical Center (LUMC) Leiden The Netherlands; ^3^ The Jackson Laboratory for Mammalian Genetics Bar Harbor Maine USA; ^4^ Sanofi Bridgewater New Jersey USA; ^5^ Immunai New York New York USA; ^6^ Department of Genetics and Genome Sciences University of Connecticut Health Center Farmington Connecticut USA

**Keywords:** aging, epigenome, health, immune aging, inflammation, longevity, mouse, transcriptome

## Abstract

Diverse mouse strains have different health and life spans, mimicking the diversity among humans. To capture conserved aging signatures, we studied long‐lived C57BL/6J and short‐lived NZO/HILtJ mouse strains by profiling transcriptomes and epigenomes of immune cells from peripheral blood and the spleen from young and old mice. Transcriptional activation of the AP‐1 transcription factor complex, particularly *Fos*, *Junb*, and *Jun* genes, was the most significant and conserved aging signature across tissues and strains. ATAC‐seq data analyses showed that the chromatin around these genes was more accessible with age and there were significantly more binding sites for these TFs with age across all studied tissues, targeting pro‐inflammatory molecules including *Il6*. Age‐related increases in binding sites of JUN and FOS factors were also conserved in human peripheral blood ATAC‐seq data. Single‐cell RNA‐seq data from the mouse aging cell atlas Tabula Muris Senis showed that the expression of these genes increased with age in B, T, NK cells, and macrophages, with macrophages from old mice expressing these molecules more abundantly than other cells. Functional data showed that upon myeloid cell activation *via* poly(I:C), the levels of JUN protein and its binding activity increased more significantly in spleen cells from old compared to young mice. In addition, upon activation, old cells produced more IL6 compared to young cells. In sum, we showed that the aging‐related transcriptional activation of *Jun and Fos* family members in AP‐1 complex is conserved across immune tissues and long‐ and short‐living mouse strains, possibly contributing to increased inflammation with age.

## Graphical abstract

Here we show that transcriptional activation of the AP‐1 transcription factor (TF) complex members, particularly Fos, Junb, and Jun genes, is the most significant and conserved aging signature across immune cells and tissues in both short (NZO) and long‐living (B6) mice strains. Genomic and functional data showed that chromatin accessibility levels around these genes and the binding activity of their TFs increase with age. These TFs target pro‐inflammatory molecules (e.g., Il6); therefore contributing to increased inflammation with age.

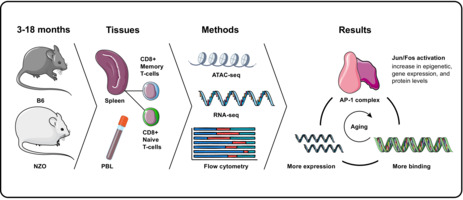

**Abbreviations**
AP‐1: activator protein 1
B6: black 6
DA: differentially accessible
DE: differentially expressed
EM: effector memory
MAG: magnitude of association of genes
NK: natural killer
NZO: New Zealand obese
PBL: peripheral blood leukocytes
PBMC: periheral blood mononuclear cells
PC: principal component
T2D: Type 2 diabetes
TF: transcription factor
TIV: trivalent influenza vaccine
TSS: transcription start site

## 1 INTRODUCTION

Age‐related changes in the immune system reduce older individuals’ ability to generate protective responses to immunological threats and lead to increases in diseases and infections (Boraschi et al., 2013; Weyand & Goronzy, 2016). Increased inflammation with age (i.e., inflammaging) is one of the hallmarks of immune system aging that is conserved across human and mouse as well as across tissues and strains (Benayoun et al., 2019); however, the drivers of this aging signature are mostly unknown (Mogilenko et al., 2021). Human immune aging studies have mostly been limited to blood since it is easy to access and gives an opportunity to study the status of the peripheral immune system with minimal invasiveness. These studies have uncovered significant age‐related changes in gene expression levels in whole blood, as well as in blood‐derived peripheral mononuclear cells (PBMCs) and sorted immune cells (Moskowitz et al., 2017; Peters et al., 2015; Ucar et al., 2017). Through genomic profiling, we and others have uncovered that pro‐inflammatory molecules are activated with age, whereas molecules related to T‐cell homeostasis and signaling are downregulated (Goronzy et al., 2018; Márquez et al., 2020; Mogilenko et al., 2021; Moskowitz et al., 2017; Nikolich‐Žugich, 2008; Ucar et al., 2017). Although these studies have described significant age‐related changes in transcriptional regulatory programs of immune cells, including the activation of pro‐inflammatory programs, they have not pinpointed potential upstream regulators of these genomic alterations.

Mouse models are essential in aging research for establishing age‐related changes in various tissues, drivers of these changes, and ways to delay or reverse these changes (Folgueras et al., 2018). Most aging studies utilize the long‐living laboratory strain C57BL/6J (B6) with a median life span of 901 days for males and 866 days for females (Yuan et al., 2009). However, there is significant diversity among mouse strains in terms of health and life span that could be exploited to uncover signatures of aging conserved among diverse human populations. An example of such a strain is New Zealand Obese (NZO/HILtJ), which resembles human aging in multiple aspects. First, similar to humans, NZO females live longer than males (Austad & Fischer, 2016), where the median lifespan is 576 days for females and 423 days for males (Yuan et al., 2009). Second, NZO mice develop obesity and hence are used as models in Type 2 diabetes (T2D), insulin resistance, and obesity (Melez et al., 1980) research. Obesity is an epidemic in human populations and a factor that accelerates the mechanisms of aging, significantly contributing to unhealthy aging. Thus, NZO and B6 represent “unhealthy aging” and “healthy aging” models, respectively, enabling us to uncover biomarkers of aging that are consistent despite differences in genetic backgrounds and life and health spans.

In these two strains, we characterized the effects of age on the immune system by profiling peripheral blood leukocytes (PBL) and the cells from the spleen, the largest lymphoid organ, both of which harbor post‐differentiated immune cells affected by aging. We also profiled naive and memory CD8^+^ T cells sorted from the spleen, given the importance of CD8^+^ T cells in human aging (Moskowitz et al., 2017; Ucar et al., 2017). In these strains and tissues, we studied the effects of age on the transcriptome (*via* RNA‐seq), epigenome (*via* ATAC‐seq), and cell compositions (*via* flow cytometry) by generating and comparing data from young (3 months) and old (18 months) animals. Together, our data uncovered transcriptional activation of Jun and Fos members of the AP‐1 complex with age as the most conserved biomarker of immune aging in long‐ and short‐living mouse strains and across spleen and blood immune cells. Our results, including functional data on JUN binding and IL6 production, suggest that AP‐1 complex activation may be a driver of inflammaging. Data from these two mouse strains and human PBMCs can be queried at: http://immune‐aging.jax.org.

## 2 RESULTS

### 2.1 Profiling blood and spleen immune cells of young and old long‐lived B6 and short‐lived NZO mice

**FIGURE 1:**
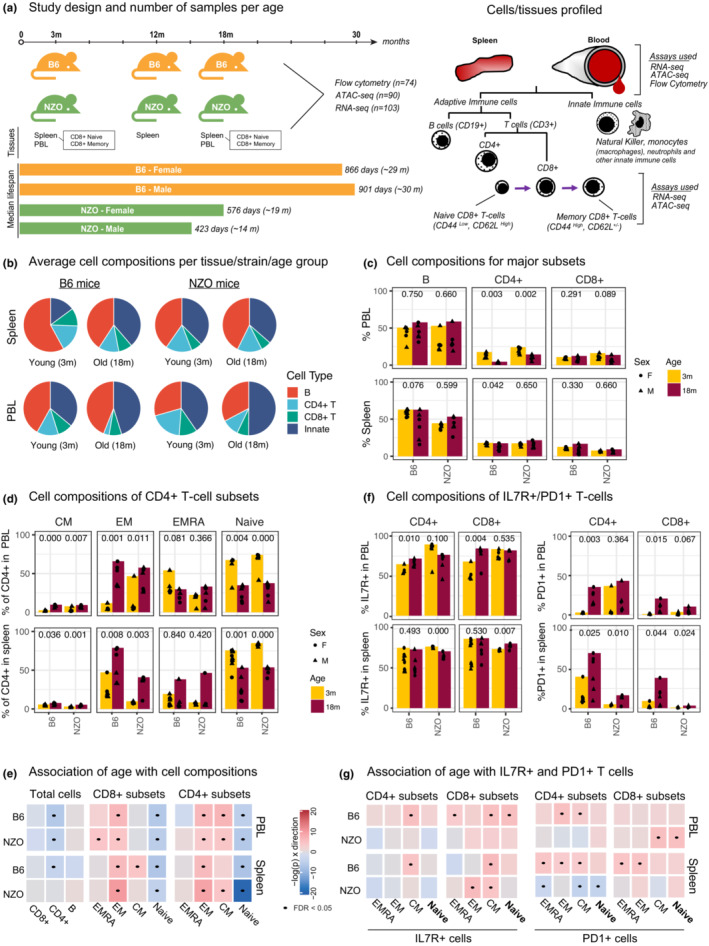
(a) Schematic of study design. PBL and spleen were collected from 3‐ and 18‐month‐old mice, and CD8^+^ T cells were sorted from spleen. We also collected and profiled spleen from 12‐month‐old mice. From these samples, ATAC‐seq, RNA‐seq, and flow cytometry data were generated. Right panel: markers used for sorting/staining in our study. (b) Overall cell composition pie charts for PBL and spleen from young and old mice for B, CD4^+^ T, and CD8^+^ T cells. The remaining cells were designated as “innate.” (c) Changes in the cellular composition of major cell types in mouse spleen and PBL with age (*x*‐axis represents age in months, and each dot represents an animal). The jitter around points is for plotting purposes to avoid overlapping dots. (d) Similar to (c) for CD4^+^ T‐cell subsets (CM: Central Memory, EM: Effector Memory, EMRA: Effector Memory re‐expressing *CD45RA*). (e) Summary for the association of age with a given cell type using linear models. Each cell type was colored according to the slope of change per unit of age (in months) and the direction of the slope—red for positive (increase with age) and blue for negative (decrease with age) slopes, with darker colors indicating steeper slope (i.e., more significant changes). Significant associations (FDR 5%) are marked with a dot. (f) Cell composition changes of IL7R^+^ and PD1^+^ T‐cell subsets. (g) Linear models that associate IL7R^+^ and PD1^+^ T‐cell percentages to age similar to (e). *p*‐values in c‐d‐f are calculated using unpaired *t*‐test.

To comprehensively study the effects of aging on the mouse immune system, we generated flow cytometry, RNA‐seq, and ATAC‐seq data from circulating immune cells (PBLs) and from the spleen in B6 and NZO strains. These strains were selected as representatives of each end of the longevity spectrum of laboratory mice to mimic the heterogeneity in individuals’ life spans and to uncover conserved immune aging signatures in long and short‐living animals. PBL and spleen samples were collected from young (3 months) and old (18 months) mice, which also allows direct age comparison to existing single‐cell mouse transcriptomic data from the Tabula Muris Senis atlas (SanMiguel et al., 2020) (Figure 1a). CD8^+^ T cells are significantly affected with age among the human peripheral immune cells (Mogilenko et al., 2021; Ucar et al., 2017), where aging affects both naive and memory compartments (Ucar et al., 2017). For this reason, we also sorted and profiled epigenomes and transcriptomes of naive and memory CD8^+^ T cells from the spleen to study whether age‐related changes in the human CD8^+^ cells (Ucar et al., 2017) also exist in mice (markers used are summarized in Figure 1a right panel). Samples that passed quality control (QC) were used in downstream analyses: 74 flow cytometry samples (39 B6, 35 NZO), 103 RNA‐seq samples (49 B6, 54 NZO), and 90 ATAC‐seq samples (38 B6, 52 NZO) (summarized in Table S1, Figure S1A). After QC and filtering, 96,623 consensus ATAC‐seq peaks from all cell/tissue types and 18,294 expressed genes from RNA‐seq samples were used in downstream analyses. Using flow cytometry, we characterized PBL and spleen cell compositions for a total of 36 cell types including total B, CD8^+^, and CD4^+^ T cells and their subsets (Table S2).

Principal component analysis (PCA) revealed that RNA‐seq, ATAC‐seq, and flow cytometry samples were first separated by tissue/cell type and then by strain as expected (Figure S1B–D). We quantified how much variation is attributable to meta‐data in each modality using principal variance component analysis (PVCA) (Li, Bushel, et al., 2009). Tissue/cell type explained most of the variation in both RNA‐seq (~55%) and ATAC‐seq (~41%) data, followed by strain differences (16% in RNA‐seq, 19% in ATAC‐seq) (Figure S1E). Age explained 20% of the variation in the flow cytometry data, due to significant remodeling of PBL and spleen cell compositions with age, whereas it explained around 5% of the variation in RNA‐seq and ATAC‐seq data. Only <2% of the variation in each modality was attributable to biological sex (Figure S1E). These data and analyses suggest that cell type and strain are the main drivers of variation in mouse genomics data, followed by the age of the animals.

### 2.2 Naive T‐cell decline is the most significant and conserved age‐related cell compositional change

Using flow cytometry, we first quantified the proportion of B, CD4^+^, and CD8^+^ T cells within the spleen and PBL (Table S2). The myeloid compartment (granulocytes, monocytes, and dendritic cells—DCs) and NK cells constitute the remainder of the cells (henceforth labeled as “innate” cells). The T‐cell subsets were further stratified into effector memory (EM, CD44^high^ CD62L^−^), effector (EMRA, CD44^low^ CD62L^−^), central memory (CM, CD44^high^ CD62L^+^), and naive (CD44^low^ CD62L^+^) cells (Figure S2A). Given their importance in T‐cell homeostasis, signaling (Nikolich‐Žugich, 2008; Tan et al., 2002), and in human aging (Ucar et al., 2017), we also quantified IL7R^+^ and PD1^+^ cells (Mogilenko et al., 2021; Pauken & Wherry, 2015).

The majority of mouse spleen cells (45%) and PBLs (37%) were composed of B cells (Figure 1b). Total B and CD8^+^ T‐percentages did not change significantly with age, whereas CD4^+^ T cells declined significantly in PBL (Figure 1c). Regarding T‐cell subsets, the most significant change was the decline of naive T cells, observed in both B6 and NZO, in both tissues (PBL, spleen), and in both CD4^+^ and CD8^+^ compartments (Figure 1d, Figure S3A). We also detected significant increases in EM populations in both compartments. Regression models that associate age with the percentage of each cell type confirmed that declines in naive CD4^+^ and CD8^+^ T cells and increases in EM CD4^+^ and CD8^+^ T cells were the most significant and conserved age‐related changes in cell composition (Figure 1e). Interestingly, these cell compositional changes were similar between the two tissues (PBL, spleen) and between the two strains despite the significant difference in their life spans. Previous mouse studies similarly reported declines in naive T cells and increases in memory T cells both in spleen (Pinchuk & Filipov, 2008) and blood (Chen et al., 2002). Naive T‐cell decline with age partially stems from insufficient homeostatic proliferation due to thymus shrinkage and continuous activation of naive T cells (Zhang et al., 2021).

The majority of CD4^+^ and CD8^+^ T cells were IL7R^+^ in both tissues and strains, and there was a slight increase in IL7R^+^ cells in naive and memory T‐cell subsets, especially in the central memory (CM) component (Figure 1g, Figure S3B). On the contrary, percentages of PD1^+^ T cells increased with age in T‐cell subsets (Figure 1f,g). The increases in PD1^+^ cell percentages were more significant in long‐lived B6 compared to short‐lived NZO at 18 months of age (Figure 1g, Figure S3B), suggesting that the increases in PD1^+^ T‐cell percentages might not strictly relate to the longevity of the organism. Together, these data and analyses showed that PBL and spleen cell compositions significantly change with age, and most of these changes are conserved between the two mouse strains, where declines in naive T cells and increases in EM cells are the most significant and conserved age‐related changes.

### 2.3 Transcriptional activation of Jun/Fos members of the AP‐1 complex is a conserved aging signature

**FIGURE 2:**
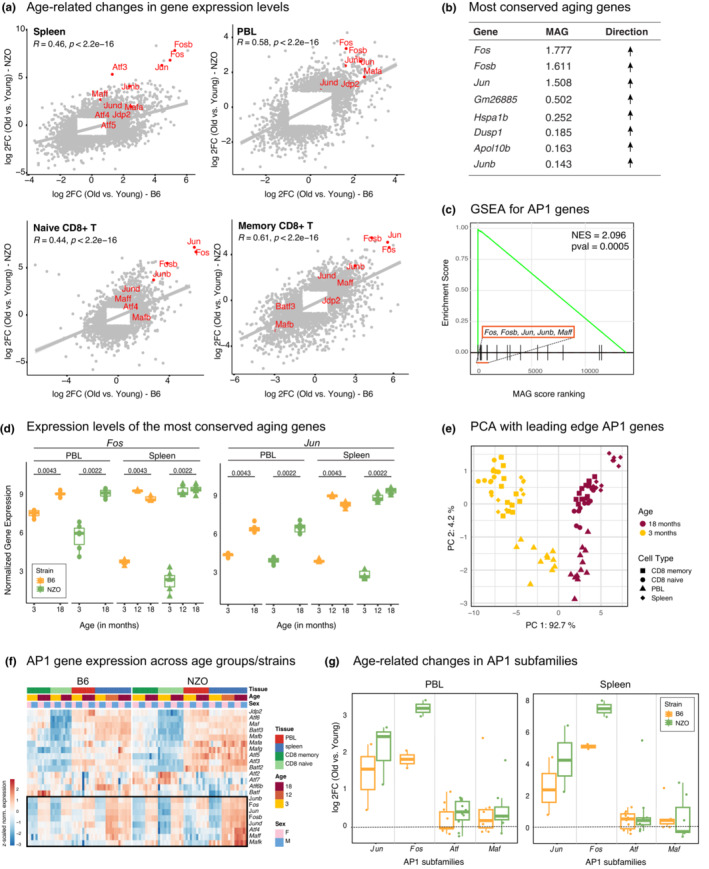
(a) Comparison of fold changes for differentially expressed genes (FDR <0.05) in two strains in each tissue/cell type. Note that transcriptional changes with age are highly conserved across strains. AP‐1 complex genes are highlighted in red. (b) Top conserved aging genes (sorted with respect to MAG scores) and the direction of changes with age (upregulated in all cases). (c) Geneset enrichment analysis (GSEA) for MAG‐ranked genes using AP‐1 complex members as the gene set. Note that top MAG genes are enriched in the AP‐1 complex. Leading age genes are denoted. The green line represents the Running Enrichment Score for GSEA, the black lines show where the members of the AP‐1 complex appear in the ranked list of genes, and the red box indicates the leading‐edge subset. (d) *Fos* and *Jun* expression levels at different ages. Expression values were log(*cpm*) normalized; p‐values are calculated using Wilcoxon rank sum test. (e) PCA plot using the expression of leading‐edge genes (*Fos*, *Fosb*, *Jun*, *Junb*, *Maff*), note that these five genes clearly separate old samples (dark red) from young ones (yellow) across tissues and strains. Note that the first PC in these data is associated with age across tissues and strains. (f) Gene expression levels of AP‐1 complex genes across all samples. The black rectangle shows the AP‐1 member genes that are consistently upregulated with age. Libraries were normalized using *cpm* function from edgeR package, and the gene expression values were z‐transformed. (g) Log2 fold changes (positive values refer to upregulation with age) of AP‐1 subunits in PBL (left) and spleen (right). Every dot maps to a gene in the corresponding AP‐1 subunit. Note that Jun/Fos members are the most strongly affected with aging, all of which are significantly upregulated in both B6 and NZO.

We conducted differential gene expression analyses between old and young animals for all studied cells/tissues: spleen, PBL, and naive and memory CD8^+^ T cells (Table S3, FDR = 0.05 and |logFC| > 1). In the spleen tissue, 1960 genes were differentially expressed (DE) in B6 and 1795 in NZO, 675 of which were shared between strains. In PBL, we detected 455 DE genes in B6 and 862 DE genes in NZO, whereas in naive CD8^+^ T cells, 826 and 1718 genes were DE with age in B6 and NZO, respectively. Functional annotation of DE genes using immune modules (Chaussabel et al., 2008) and cell‐specific gene sets from human PBMC scRNA‐seq data showed that genes associated with inflammation and the myeloid lineage were activated with age in both strains in PBL and spleen (Table S4, Figure S4A), whereas naive T cell‐related genes were inactivated with age—in alignment with cell compositional changes. The most significantly upregulated genes in memory CD8^+^ T cells included age‐associated T‐cell (T^aa^) markers (Mogilenko et al., 2021), notably *Gzmk* and *Ifng*, in both strains; and memory CD8^+^ DE genes were enriched in marker genes associated with this cell type (Figure S4B, C). To uncover the most conserved signatures of immune aging, we first compared age‐related gene expression changes between the two strains within each tissue, which revealed a positive and significant correlation suggesting that most age‐related changes are conserved (correlation coefficient *R* = 0.44 to 0.61, *p* < 2.2.e−16 for all tissues) (Figure 2a). To uncover genes that are the most significantly and robustly associated with aging, we calculated a novel similarity score based on the magnitude of association of genes (MAG) to a phenotype, which was originally developed to assess the similarity between diseases (Luo et al., 2021). First, we calculated MAG scores for each gene based on their association with aging across the 4 studied cells/tissues as well as the two strains (Figure 2b, Table S5). Interestingly, the top three most consistently and significantly aging‐associated genes (all upregulated) were *Fos*, *Fosb*, and *Jun*, which are members of the Activator Protein‐1 (AP‐1) complex. Gene Set Enrichment analyses of genes sorted with respect to their MAG score confirmed that AP‐1 genes were significantly enriched among the most conserved aging genes (NES = 2.096, *p* = 0.005) and showed that 5 genes contributed the most to this enrichment: *Fos*, *Fosb*, *Jun*, *Junb*, and *Maff* (Figure 2c). In alignment, DE genes for each cell/tissue type were also enriched for AP‐1 genes (Figure S4C). For example, the expression of *Fos* gene increased significantly across all 4 cell types/tissues in both strains: ~7‐fold and ~5‐fold increases in NZO and B6 spleen, respectively (Figure 2d, Table S3). To study the timing of age‐related changes, we have also collected spleen samples from both strains at 12 months. Interestingly, these data showed that these genes were activated at 12 months, suggesting that some of these changes might start in middle‐aged animals (corresponding to 40–50 years in humans) (Figure 2d). Overall, activation of Jun/Fos family genes was more significant in shorter‐living NZO animals in both tissues. We conducted PCA analyses using only these 5 genes (Figure 2e) and observed that the biggest variation in these data (captured in PC1, explains 93% of the variation) is associated with aging, since young and old samples clearly separated in PC1 across all 4 cell/tissue types. PC2 was associated with tissue/cellular origin. These results reinforce the strong association of these genes with aging.

AP‐1 is a protein complex that regulates transcriptional responses to diverse stimuli including stress and infections (Foletta et al., 1998; Karin et al., 1997; Wagner, 2001). Structurally AP‐1 is a heterodimer composed of proteins that belong to different subfamilies including Fos (Fos, FosB, Fra‐1, Fra‐2), Jun (Jun, JunB, and JunD), and ATF (ATFa, ATF‐2, and ATF‐3) families (Hess et al., 2004; Wu et al., 2021). Closer analyses of distinct protein families in the AP‐1 complex showed that genes in the JUN/FOS subfamilies were specifically and most significantly activated with age, whereas members of ATF and MAF subfamilies were mostly not associated with age (Figure 2f,g, Figure S4D, Tables S3 and S6). Together, these data show that the activation of Jun/Fos members in the AP‐1 complex is a highly conserved aging signature detected in both strains (B6, NZO) and tissues (PBL, spleen), across all four cell/tissue types, including T‐cell subsets (naive and memory CD8^+^).

Although age‐related changes are significantly conserved between strains (Figure S4E, F), we detected and studied molecules that are differentially expressed with age only in one strain (i.e., passing the statistical significance threshold only in one strain) and we annotated these genes using WikiPathways and GO terms (Tables S7 and S8). Genes that are upregulated specifically in NZO were enriched for oxidative phosphorylation, mitochondrial translation (genes encoding mitochondrial ribosomal proteins), NFKB (*Rela*, *Relb*, *Nfkbia* in spleen), and insulin signaling (*Igf1r*, *Irs2* in PBL), as well as inflammation (e.g., *Tnf*, *Nlrp3*, and caspases in spleen). These data suggest that NZO animals experience several hallmarks of aging (inflammaging, mitochondrial dysfunction, deregulated nutrient sensing) more significantly than the B6 strain at the same age, which is in alignment with the shorter lifespan of NZO animals and their increased risk for T2D and obesity.

### 2.4 Chromatin accessibility at the Jun/Fos promoters and at their binding sites increases with age

**FIGURE 3:**
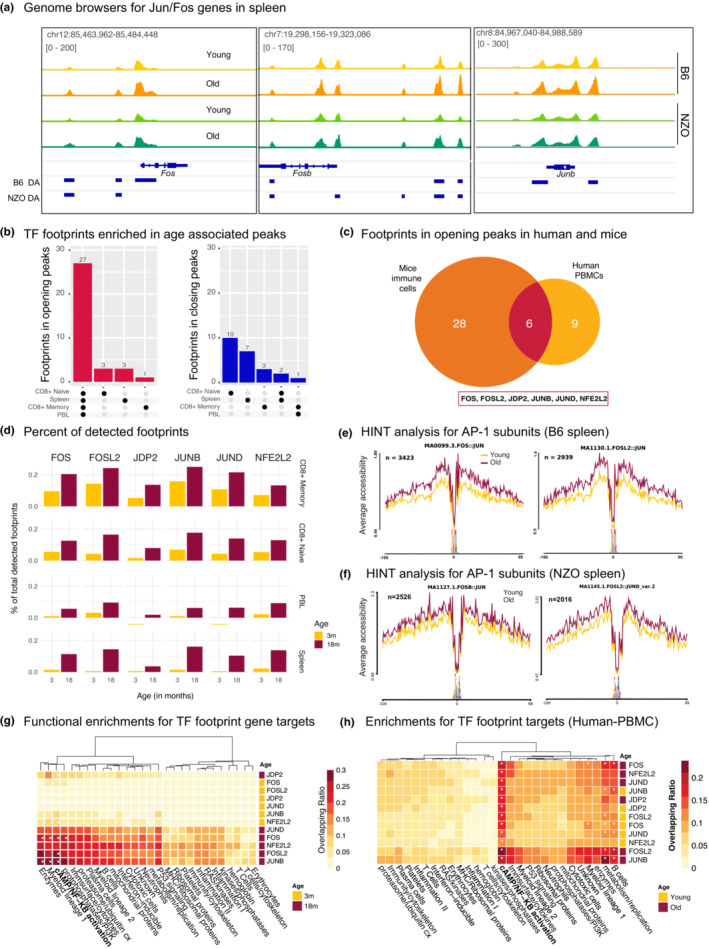
(a) Genome browser tracks that show chromatin accessibility data at the *Fos*, *Fosb*, *Junb* genes in spleen cells. Note that in both strains there is increased chromatin accessibility in older spleen cells at these loci. Bars underneath represent differentially accessible (DA) peaks in B6 and NZO strains loci. All genome browser tracks are scaled to the same value which is depicted on the left top corner. (b) Upset plots that summarizes overlaps among enriched transcription factor (TF) footprints across tissue/cell types (adjusted *q* < 0.05 using BiFET). Left panel: 27 out of 34 TF footprints enriched within opening peaks are shared across tissue/cell types. Right panel: TF footprints enriched within closing peaks are mostly cell type specific. (c) Overlap of footprints enriched in opening peaks in human and mice. 6 proteins were common including five AP‐1 members (FOS, FOSL2, JDP2, JUNB, JUND), and a co‐factor of the complex NFE2L2. (d) Percent of footprints detected for the shared 6 TFs in each age group and cell type. Counts are normalized with respect to the total number of footprints detected. Note that there are more binding events for these TFs with age. (e, f) Chromatin accessibility levels at the JUN/FOS footprints for (e) B6 spleen and (f) NZO spleen cells. Note that in older spleen cells, these sites are more accessible. The number of footprints used in each analysis is listed at the top left corner. Functional enrichment of gene targets for conserved age‐associated TFs using immune modules in B6 PBL (g) and human PBMCs (h). Values show the percent of overlap between gene targets and module genes; significant enrichments are indicated with an asterisk (FDR < 0.05). Note the increase in NFkB pathway in older samples.

We uncovered differentially accessible (DA) ATAC‐seq peaks with age and mapped these to the closest transcription start site (TSS) (Table S9, FDR = 0.05 and |logFC| > 1, Tables S10 and S11 for strain‐specific DA peaks). As expected, genes associated with opening peaks overlapped significantly with genes upregulated with age, whereas closing peak genes overlapped significantly with genes downregulated with age (Figure S5A). Functional enrichment of DA peaks was also in alignment with differential expression results, where the most consistent and strong age‐associated signal was the epigenetic activation of pro‐inflammatory molecules (Figure S5B). We detected significant increases in chromatin accessibility levels around the promoters of Jun/Fos family genes in older samples; this age‐related epigenetic activation of Jun/Fos genes was statistically significant and was conserved across strains, tissues, and cell types (Figure 3a, Figure S5C for spleen, Figure S6 for PBL and CD8^+^ subsets).

To uncover whether binding events of Jun/Fos TFs are also affected, we conducted footprinting analyses in ATAC‐seq data, which *in silico* infers TF binding events by integrating chromatin accessibility patterns with the underlying TF sequence motifs (Gusmao et al., 2016). First, we called TF footprints (Sherwood et al., 2014) in mouse samples from pooled young and old animals in four different tissue/cell types using known TF motifs (Khan et al., 2018), as well as in human PBMCs from young and old subjects (Márquez et al., 2020; Ucar et al., 2017). Samples were pooled to increase the depth of sequencing, increasing the number and quality of detected footprints (Youn et al., 2019). Next, we identified TF footprints enriched in peaks that were activated (i.e., opening) or inactivated (i.e., closing) with age in each tissue/cell type (Youn et al., 2019). Interestingly, TF footprints enriched in opening peaks were largely shared across the 4 cell types in mice (27 out of 34 were detected in all studied cells/tissues), in contrast TF footprints enriched in closing peaks were cell/tissue type‐specific (Figure 3b). Among the footprints that were enriched in opening peaks with age, six were also enriched in opening peaks from human PBMCs (Figure 3c, Table S12). Remarkably, this included five AP‐1 complex members (FOS, FOSL2, JDP2, JUNB, JUND) as well as NFE2L2 (i.e., NRF2), a protein that interacts with ‐JUN and contributes to the regulation of the NLRP3 inflammasome (Ahmed et al., 2017). The baseline activated JUN/FOS status in older mice can be an important modulating element of the biological responses of the aging immune cells. Interestingly, the chromatin accessibility levels around the promoters of these TFs increased in men with aging—inferred from PBMC RNA‐seq data from our previous study (Ucar et al., 2017); notably older men experience accelerated myeloid activation and “inflammaging” compared to older women (Figure S7A). In alignment with the footprinting results, motif enrichment analyses confirmed that opening peaks with age were enriched in motifs of these TFs, particularly JUN/FOS families both in human and mouse immune cells (Figure S7B, Table S13).

In addition to the enrichment of these six TFs among opening peaks, footprints for these TFs made up a larger proportion of all detected footprints in older samples across the four tissues (Figure 3d, Table S14). We also compared the cleavage profiles from the ATAC‐seq libraries in young and old mice, to get insights into chromatin accessibility profiles at the binding sites of JUN/FOS TFs (Gusmao et al., 2014, 2016). Chromatin around their binding sites was more accessible in cells from old mice compared to cells from young mice, which was observed in tissues (PBL, spleen) (Figure 3e,f) and in CD8^+^ subsets in both strains (Figure S7C, D). To understand which cellular functions are modulated by the increased “binding” of these TFs, we identified their gene targets based on the distance to TSS. These gene targets included members of the Nf‐KB pathway (*Rel*, *Rela*, *Nfkbiz*), pro‐inflammatory cytokines and chemokines (*Il1b*, *Il6*, *Il15*, *Cxcl10*), genes expressed by activated myeloid (*Cd86*, *Cd44*, *Il7r*, *S100a11*) and lymphoid cells (*Cd44*, *Cd28*) (Lawlor et al., 2021), cytotoxic molecules (*Gzmk*, *Gzmb*, *Klrg1*), and plasma cell marker *Cd38* (Table S15). Our results suggest that these TFs modulate important immune responses in both innate and adaptive immune cells. These gene targets were functionally enriched in pro‐inflammatory immune modules and pathways (e.g., Nf‐KB activation) across mouse tissues and strains (Figure 3g, Figure S7E). Similarly, in human PBMCs, TF gene targets included pro‐inflammatory (*FOSL2*, *LMNA*, *CASP8*, *NFKBIZ*), cytotoxic (*GNLY*, *PRF1*, *GZMB*), and activated cell markers (*CD44*, *IL7R*) (Lawlor et al., 2021), and were enriched in pro‐inflammatory pathways/modules (Figure 3h, Table S16). These results support the previously reported cross‐regulation of AP‐1 complex and NF‐kB pathways in myeloid cells (Fujioka et al., 2004; Ji et al., 2019) and provide further insight into other pathways and functions potentially regulated by these TFs in both myeloid and lymphoid cells. Together, our findings nominate increased JUN/FOS TF activity as a conserved biomarker of immune system aging involved in regulating pro‐inflammatory and effector molecule functions, thereby potentially contributing to inflammaging.

### 2.5 Expression of *Jun/Fos* increases with age across all immune cell types from the spleen

**FIGURE 4:**
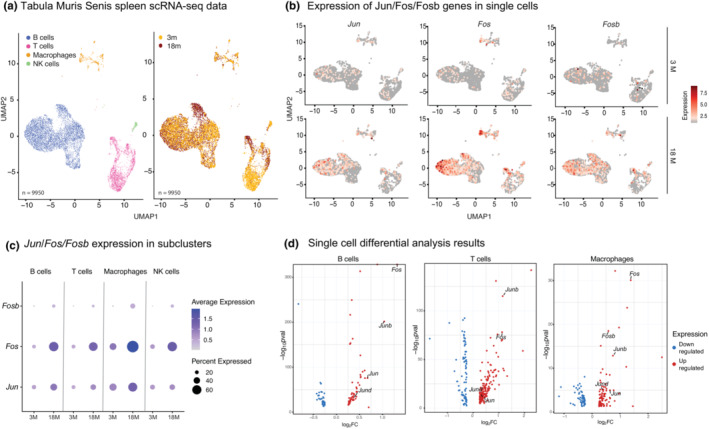
(a) Left: UMAP for the Tabula Muris Senis spleen data using young (3‐month, 3 m) and old (18‐month, 18 m) B6 mice spleen (*n* = 2, per age group). Cell types that have <100 cells were removed from the downstream analyses, resulting in four major cell types; B cells, macrophages, natural killer (NK) cells, and T cells (*n* = 9950 cells). Right: Cells are annotated with respect to the age of the animals. (b) The expression levels of *Jun*, *Fos*, *Fosb genes* in log‐normalized scale in young (3 m, top) and old cells (18 m, bottom). Note the increase in expression of these molecules across all cell types. (c) Dotplots summarizing the expression of Jun, Fos, and Fosb genes in immune cell subsets. The size of the dot represents the proportion of cells expressing that specific gene, and the color of the dot represents the level of expression. Note the increase in expression/abundance of these molecules with age across all subsets. (d) Volcano plots that summarize differential expression result from scRNA‐seq data. Red dots represent significantly upregulated genes; blue dots represent significantly downregulated genes (*p* = 0.05). Note that Jun and Fos family members are significantly upregulated across cell types.

To uncover whether age‐related transcriptional activation of Jun/Fos genes stems from specific cell types, we reanalyzed single‐cell RNA‐seq data from the Tabula Muris Senis consortium (Consortium, 2020) using spleen cells from young (3 months) and old (18 months) B6 mice. We annotated spleen cells as B, T, NK cells, or macrophages using known marker genes (Figure 4a). In alignment with our flow cytometry data (Figure 1b), the majority of spleen cells were composed of B cells, followed by T cells and innate immune cells (Figure S8A, B). Next, we studied the activation of the most conserved aging genes (*Jun/Fos/Fosb*) (Figure 2b) in these single cells. The expression of these molecules increased with age across all immune cell types (Figure 4b,c, Figure S8C). Their activation with age was observed at both the level of expression and the frequency of expression (Figure 4c, Figure S8C). For example, older B, T, and macrophage cells expressed Fos and Jun at significantly higher levels than their younger counterparts. In addition, the percentage of cells that expressed these molecules also increased. For example, ~78% of older macrophages expressed *Fos*, compared to 43% of young macrophages (Figure S8C). We also conducted differential expression analyses at the single‐cell level between young and old mice for T, B, macrophage, and NK cells using MAST. These results also confirmed that these genes are significantly upregulated with age across the four cell types studied here (Figure 4d, Table S17). Together, these data showed that age‐related activation of Jun/Fos family genes are a conserved signature of cellular aging across different immune cell types, where macrophages express these molecules most significantly, potentially to regulate increased inflammatory responses with age.

### 2.6 JUN protein production and binding increase with age upon stimulation *via* Toll‐like receptors

**FIGURE 5:**
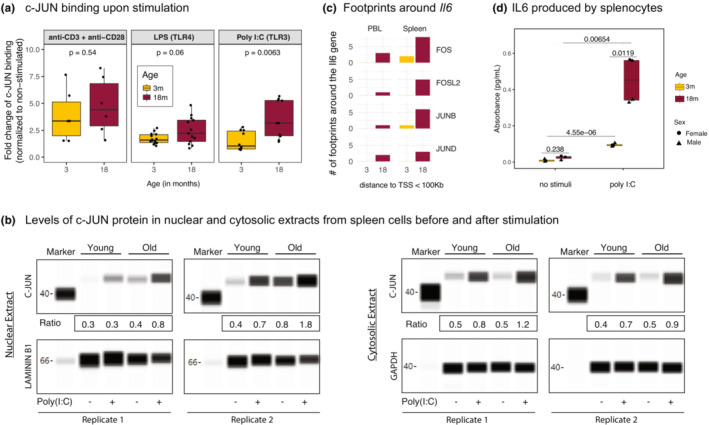
(a) JUN binding levels upon different stimuli (anti‐CD3^+^ anti‐CD28, LPS, and poly(I:C)) for young and old B6 mice spleen. JUN binding levels were normalized to the non‐stimulated samples. Note the more pronounced increase in JUN binding in old spleen cells. *p*‐values are calculated using the Wilcoxon rank sum test. (b) Western blot of c‐JUN proteins from the nuclear and cytosolic extracts of male B6 mice with and without poly(I:C) stimulation. Note that c‐JUN levels increase with age in nuclear extracts with age both at baseline and upon poly(I:C) stimulation. The ratio of JUN protein to the housekeeping protein (LAMININ B1 for nuclear extract and GAPDH for cytosol) was quantified and included under the gels (labelled Ratio). (c) Number of footprints detected around the *Il6* gene (100 kb upstream and 100 kb downstream of the TSS from each side) in B6 PBL and spleen for JUN/FOS TFs. Note the increased binding events around this molecule with age. (d) IL6 cytokine levels secreted by splenocytes of young (3 months) and old (18 months) B6 mice spleen. Note the increased production of IL6 with age upon poly(I:C) stimulation. *p*‐values are calculated using unpaired *t*‐test.

An increase in the transcription of genes that form complexes may not necessarily indicate increased function. This is particularly true for the AP‐1 protein complex, being a heterodimer composed of FOS, JUN, and ATF protein families and whose activity depends on the formation of the complex and post‐translational modifications such as phosphorylation (Angel & Karin, 1991). To understand whether age‐related transcriptional/epigenetic activation of *Jun/Fos* affects protein levels and AP‐1 function, we studied JUN protein expression *via* Western blotting and protein binding *via* a JUN transcription factor functional assay ELISA kit (Table S18). We used splenocytes and not sorted immune cell subsets for these assays since a robust immune response requires crosstalk between different immune cells (Srivastava et al., 2019; Tan et al., 2014). First, we quantified JUN binding activity in nuclear extracts from the B6 spleen cells upon stimulation using (1) anti‐CD3/anti‐CD28 to stimulate T cells; (2) LPS to stimulate B cells and monocytes *via* TLR4; and (3) poly(I:C) to stimulate monocytes *via* TLR3. The JUN binding activity level did not significantly change between age groups upon T‐cell stimulation; however, it significantly increased with age upon TLR‐mediated stimulation (Figure 5a), particularly upon poly(I:C) stimulation, which activates monocytes (*p* = 0.0063). TLR3 activation by poly(I:C) has been shown to regulate inflammatory responses in tissues (Stowell et al., 2009). To complement the binding assay, we also quantified the level of JUN protein in nuclear and cytosolic fractions of the spleen before and after stimulation by Western blotting for JUN and loading markers LamininB1 for nuclear extracts and GAPDH for cytosolic extracts. In the nuclear extract, even before the activation of cells, splenocytes from older animals had more JUN protein compared to those from younger animals (Figure 5b). Upon poly(I:C) stimulation, nuclear and cytosolic JUN protein levels increased in both young and old splenocytes as expected; however, the increases were more significant in older cells (Figure 5b), in alignment with their transcriptional and epigenetic activation (Figures 2 and 3). Together, our data demonstrate that when activated *via* TLR3 spleen cells from older mice have increased JUN protein and JUN binding compared to cells from younger mice.

In pooled splenocytes, poly(I:C) stimulation led to the greatest increase in JUN binding, which stimulates monocytes *via* TLR3. Monocytes govern the innate immune responses by initiating inflammation, through the production of pro‐inflammatory cytokines including IL6. AP‐1 complex components are important in regulating inflammation (Shaulian & Karin, 2002); however, their role in inflammaging is unknown. To further explore this connection, we focused on the IL6 cytokine—a canonical marker of inflammaging (Franceschi & Campisi, 2014). Footprinting analyses established that footprints for JUN/FOS TFs bound around the *Il6* promoter, and there were more footprints for these TFs around the *Il6* locus with age (Figure 5c). To further characterize the downstream effects of increased JUN activity with age, we studied whether there is increased IL6 protein production with age in TLR3‐activated splenocytes. For this, splenocytes were stimulated *in vitro* with poly(I:C) for 1 day and the supernatant was assayed for IL6. As expected, poly(I:C) stimulation led to increased IL6 levels both in young and in old splenocytes; however, this inflammatory response was significantly higher in older cells with age (*p* = 0.00654) (Figure 5d). Interestingly, spleen cells from older females produced significantly more IL6 than spleen cells from older males (*p* = 0.01, Table S19). scRNA‐seq data from spleen (Consortium, 2020) showed that the expression levels of Toll‐like receptor genes did not change significantly with age (Figure S8D), suggesting that the increased inflammatory responses upon stimulation are modulated by downstream regulators of TLR signaling, not *via* the changes in the receptor expression levels. These data confirm that aged spleen cells are more pro‐inflammatory in nature and that increased production and binding of JUN/FOS TFs with age is a potential regulator of these increased inflammatory responses.

## 3 DISCUSSION

To uncover conserved biomarkers and regulators of immune aging, we comprehensively profiled diverse immune cells and tissues in long‐living B6 and short‐living NZO mouse strains. NZO mice develop T2D and obesity and can thus be considered a model for “unhealthy aging.” Despite differences in their health and life spans, our results show significant agreement in age‐related changes between the two strains both in flow cytometry (cell compositional) and genomic (cell‐intrinsic) data. Using a novel scoring metric, we uncovered that transcriptional activation of JUN/FOS genes of the Activating Protein‐1 (AP‐1) complex—particularly the upregulation of *Jun*, *Fos*, and *Fosb* genes—is the most conserved aging signature across the two mouse strains, diverse immune tissues, and cell types. AP‐1 is a transcription factor (TF) complex that regulates gene expression programs in response to diverse stimuli, including stress, viral infections (Hess et al., 2004), and pro‐inflammatory signals in concert with the NF‐KB pathway (Fujioka et al., 2004; Ji et al., 2019; Renoux et al., 2020). ATAC‐seq data from the same tissues revealed that there is also epigenetic activation of *Jun/Fos* genes with age. Chromatin accessibility at their promoters increases with age in addition to the chromatin accessibility increases at their binding sites. Western blot data showed that there is more nuclear JUN protein in older splenocytes compared to younger ones even at baseline. Furthermore, when myeloid cells within splenocytes are stimulated *via* TLR3 (poly(I:C)), older cells produce more JUN and there is more JUN binding compared to younger cells. These data suggest that transcriptional activation of *Jun/Fos* genes, whose protein products form the AP‐1 complex, is a conserved signature of immune aging that increases the level of JUN protein production and binding with age.

Increased inflammation with age (i.e., inflammaging) is one of the hallmarks of aging observed in multiple mouse tissues (Benayoun et al., 2019) and also in human cells (Furman et al., 2019; Márquez et al., 2020), including the increased levels of IL6 in human serum (Maggio et al., 2006). Several lines of evidence indicate that the activation of *Jun/Fos* genes with age manifests most prominently in the myeloid compartment, specifically monocytes that govern the inflammatory responses. First, poly(I:C) primarily activates monocytes *via* TLR3. TLR3 is a highly conserved molecule that recognizes double‐stranded (ds) RNA associated with a viral infection and induces the activity of the interferon response and pro‐inflammatory molecules in myeloid cells (Matsumoto & Seya, 2008). Second, single‐cell RNA‐seq data from Tabula Muris Senis (Consortium, 2020) showed that, although the gene expression levels of Jun/Fos members increase significantly across all immune cell types with age, older macrophages express these molecules at higher percentages compared to other immune cell types. Footprinting analyses nevertheless suggest that these TFs can target and potentially activate important molecules across distinct immune cell types: pro‐inflammatory, cytotoxic, and effector molecules in both myeloid and lymphoid cells. Among the pro‐inflammatory molecules, we further studied IL6 as a biomarker of inflammaging. Footprinting analyses demonstrated that JUN/FOS binding events (i.e., footprints) in the vicinity of *Il6* locus increase with age. Furthermore, older spleen cells produce more IL6 upon TLR3‐stimulation compared to younger ones. Together with the increased JUN protein production and binding in these cells, these results suggest that age‐related activation of JUN/FOS TFs with age is a potential upstream regulator of inflammaging.

Blood samples are the most frequently used tissue in human aging studies; we compared age‐related changes detected in mice blood samples with the changes detected from human PBMCs. At the cell compositional level, human PBMCs were composed of more T cells and less B and innate cells compared to mice PBLs. In both humans and mice, there is a significant remodeling with age within T cells including decreases in naive and increases in memory subsets. However, in human PBMCs this decline is more striking for naive CD8^+^ T cells (Zhang et al., 2021) whereas in mice both CD4^+^ and CD8^+^ T‐cell compartments were similarly affected with age. A prominent age‐related change we previously detected in human PBMCs was the chromatin closing and reduced expression of *IL7R* and its downstream molecules in the IL7 signaling pathway (Ucar et al., 2017) that stem from the decline of IL7R^+^ CD8^+^ cells (Ucar et al., 2017). In mouse tissues and strains studied here, we detected neither the downregulation of the *Il7r* gene nor a decline in the percentages of IL7R^+^ T cells. This discordance between human and mouse CD8^+^ T‐cell aging patterns might be attributable to differences in their antigenic challenges—unlike humans, laboratory mice live in a highly controlled environment (strict diet, unchallenged immune system). In terms of the activation of AP‐1 complex members, Jun/Fos genes were not significantly upregulated with age in bulk PBMCs; however, we detected more TF footprints for these molecules with age. Furthermore, similar to mice, these TF footprints were in the vicinity of pro‐inflammatory and effector molecules in human PBMCs, suggesting that increased regulatory activity of these TFs might be a conserved signature of aging. Recent single‐cell studies from human and mouse (B6) immune cells have uncovered conserved expansion of GZMK^+^ CD8^+^ T‐cell populations with age (Mogilenko et al., 2021). In the present study, we detected the upregulation of marker genes for this T‐cell population in both strains with age, suggesting that this aging signature is conserved across different strains of mice regardless of their life span.

B6 and NZO strains exhibit differences in terms of the life span and health span of female and male animals, where NZO males live shorter and develop T2D (Figure 1a). However, we did not observe significant sex differences in the aging signatures described here, likely because the cohort was not powered to detect sex differences as this was not our main objective. Bigger cohorts will be needed to delve into differences between female and male immune system aging in different mouse strains and their potential implications for human immune aging and vaccine responses. However, we did observe an interesting sex dimorphism in the production of pro‐inflammatory IL6 upon stimulation, where female splenocytes from old B6 mice produce significantly more IL6 compared to splenocytes from old males (Figure 5d). In alignment with these results, previous studies suggested that testosterone has a suppressing effect on *Jun/Fo*s genes and this plays a role in sex differences observed in human vaccine responses (Furman, 2015; Furman et al., 2014). Another human study showed that chromatin accessibility around AP‐1 members decreases upon influenza vaccination in blood‐derived immune cells—both with trivalent influenza vaccine (TIV) and AS03‐adjuvanted H5N1 vaccine in young adults (Wimmers et al., 2021). Interestingly, this epigenetic remodeling around the AP‐1 complex members boosted responsiveness to vaccines for other viruses—Zika and Dengue (Wimmers et al., 2021). Here, we show chronic activation of AP‐1 complex members with age. In human PBMCs, AP‐1 member gene expression does not significantly increase with age, though increases were more pronounced in men. However, we detected more binding events for JUN/FOS TFs in PBMCs from older adults compared to PBMCs from younger adults, suggesting that the binding activity of these molecules might also be affected with age in human immune cells. Future studies in older adults will be important to uncover the extent of JUN/FOS remodeling with age and the contribution of AP‐1 members to reduced vaccine responsiveness in older adults.

Interestingly for spleen samples, cell compositional changes at the major population level were more modest in shorter‐living NZO compared to longer‐living B6 (total B, total T). However, within subsets of T cells, there was significant remodeling with age and naive CD4^+^ decline with age was the most significant for NZO spleen samples. On the contrary, *Jun* and *Fos* genes are significantly upregulated in the NZO spleen samples; hence, these gene expression patterns are more likely to be driven from cell‐intrinsic changes rather than cell compositional changes. This is also in alignment with the fact that we detected the upregulation of these genes in sorted naive and memory CD8^+^ T cells as well as in single‐cell RNA‐seq data from the spleen. Together, these data suggest that the activation of *Jun/Fos* genes is a cell‐intrinsic aging signature and does not always align with cell compositional changes. Although in this study, we focused on most conserved signatures of immune aging, we uncovered strain‐specific aging patterns that could serve as a resource for future studies. In sum, NZO mice experience several hallmarks of aging (inflammaging, mitochondrial dysfunction, deregulated nutrient sensing) more significantly than the B6 strain at the same age, which is in alignment with the shorter lifespan of NZO animals and their increased risk for T2D and obesity. Several genetic variants have been linked to these syndromes in the NZO mice (*Pcpt* (Pan et al., 2006), *Lepr* (Igel et al., 1997), *Ifi202b* (Vogel et al., 2012), *Abcg1* (Buchmann et al., 2007)), which might also contribute to the accelerated aging phenotype observed in the NZO mice compared to B6.

## 4 METHODS

### 4.1 Animals and housing

C57BL/6J (stock 000664) and NZO/HlLtJ (stock 002105) animals were obtained from The Jackson Laboratory and kept in individually ventilated cages with free access to food (5KOG, LabDiet) and water. The pathogen‐free room (health status report attached) was kept between 20°C and 22°C, with a 12‐h light:dark cycle. Spleen and blood samples were obtained immediately after euthanasia through cervical dislocation. The mouse study was approved by The Jackson Laboratory's Institutional Animal Care and Use Committee.

### 4.2 Flow cytometry data generation and analyses

Data are obtained from spleen and peripheral blood lymphocytes (PBLs) of C57BL/6J and NZO/HILtJ mouse strains, hereafter B6 and NZO, respectively, at ages 3, 12, and 18 months. Cells were stained with (i) CD8 FITC, Clone 53‐6.7 BD Bioscience Cat# 553031, used at 1:240 final concentration; (ii) CD3e PE, Clone 145‐2C11 eBioscience (Now ThermoFisher) cat# 12‐0031‐85, used at 1:240 final concentration; (iii) CD62L PE‐Cy7, Clone MEL‐14 Tonbo Biosciences Cat# 60‐0621‐U100, used at 1:480 final concentration; (iv) CD44 APC‐Cy7, Clone IM7 Tonbo Biosciences Cat# 25‐0441‐U100, used at 1:240 final concentration and for 30 min at 4°C. Propidium Iodide used for cell viability at 0.5 μg/ml.

Then, cells were sorted on a FACSAria II (BD Biosciences). Briefly, doublets were gated out, Viable PI‐ cells were gated, CD3e^+^, CD8^+^ cells were gated and subdivided into CD44^Low^, CD62L^High^ Naive cells and CD44^High^, CD62L^+/−^ Memory cells. Up to 50,000 cells were sorted for ATAC‐seq, remaining cells collected for other RNA preparations. Cells were collected in tubes coated with fetal bovine serum (FBS). The percentages of B, CD4, CD8, Naive CD4, Central memory CD4, Effector memory CD4, Effector memory RA CD4, Naive CD8, Central memory CD8, Effector memory CD8, Effector memory RA CD8 were measured. In addition, within each of these cell types, the percentages of IL7R^+^ and PD1^+^ cells were measured. After summing up B, CD4^+^, and CD8^+^ T cells, we labeled the rest of the percentages as monocytes since NK cells, neutrophils, and other cell types compose only ~5% of spleen and PBL. Naive and memory CD8 T cells were sorted from spleens as follows: Spleens were removed from mice and teased apart in nylon mesh bags in PBS with 2% FBS, 5 mM EDTA, and 0.02% Sodium Azide (FACS Buffer). The cells were lysed with "Gey's Buffer" (a modification of GBSS as described in the attached paper where the sodium chloride was exchanged with ammonium chloride at the same molar concentration) for 5 minutes and then washed with FACSBuffer and counted to determine concentration. Cells were stained at approximately 10^8^/mL with CD8 FITC, CD3e PE, CD62L PE‐Cy7, and CD44 APC for 30 min at 4 degrees. Cells were washed and resuspended for sorting in FACS Buffer. I sorted Naive (CD62L^+^, CD44^Low^) and Memory (CD62L^+/−^, CD44^High^) CD8^+^, CD3e^+^ cells. To quantify cell compositional changes with age, we built linear models where age is the independent variable and cell type percentage is the dependent variable. For each model, we computed the slope of change per increase in age by 1 unit and a corresponding p‐value, which is later corrected using Benjamini‐Hochberg procedure.

### 4.3 RNA isolation and QC

Total RNA was isolated from 1 million cells using the RNeasy Mini kit (Qiagen), according to the manufacturers’ protocols, including the optional DNase digest step. For samples with fewer than 1 million cells, RNA was isolated using the RNeasy Micro kit (Qiagen). Sample concentration and quality were assessed using the Nanodrop 2000 spectrophotometer (Thermo Scientific) and the RNA 6000 Nano and Pico LabChip assays (Agilent Technologies).

### 4.4 RNA library generation

30 ng of total RNA, with the addition of 6 μl ERCC Spike‐In Control Mix 1 (Ambion Thermo Fisher) diluted 1:10,000, was used for library construction. Libraries were prepared by the Genome Technologies core facility at The Jackson Laboratory using the KAPA mRNA HyperPrep Kit (KAPA Biosystems), according to the manufacturer's instructions. Briefly, the protocol entails isolation of polyA containing mRNA using oligo‐dT magnetic beads, RNA fragmentation, first and second strand cDNA synthesis, ligation of Illumina‐specific adapters containing a unique barcode sequence for each library, and PCR amplification. Libraries were checked for quality and concentration using the D5000 assay on the TapeStation (Agilent Technologies) and quantitative PCR (KAPA Biosystems), according to the manufacturer's instructions.

### 4.5 RNA sequencing

Libraries were pooled and sequenced 75 bp single‐end on the HiSeq 4000 (Illumina) using HiSeq 3000/4000 SBS Kit reagents (Illumina), targeting 40 million reads per sample. We obtained RNA‐seq data from spleen, PBL and sorted T cells (derived from spleen) of B6 and NZO mouse strains at age 3, 12, and 18 months. Single‐end RNA‐seq reads were aligned to the mouse genome (mm10) with Bowtie 2 (Langmead & Salzberg, 2012) and counts were generated with *RSEM* (Li & Dewey, 2011). To normalize the raw counts count‐per‐million (*cpm*) function from edgeR package is used and the genes that are log(cpm) < 1 and expressed less than 2 samples were excluded from rest of the analyses. For differential analysis pipeline, however, raw counts are used with the default options of edgeR package (Robinson et al., 2010), and via TMM normalization.

### 4.6 ATAC‐seq library generation

ATAC‐seq libraries were prepared using 50,000 cells, as previously described (Buenrostro et al., 2015), with the following modifications: Digitonin was added to the transposition reaction at a final concentration of 0.01%; the transposition reaction was purified using the Genomic DNA Clean & Concentrator‐10 kit (Zymo Research Corporation); PCR amplification was carried out using the Nextera DNA Library Prep (Illumina) Index Adapters, Nextera PCR Master Mix, and PCR Primer Cocktail for 10 cycles of PCR; PCR reaction was purified using 1.7× SPRI beads (Agencourt AMPure XP, Beckman Coulter). Libraries were checked for quality and concentration using the DNA High‐Sensitivity LabChip assay (Agilent Technologies) and quantitative PCR (KAPA Biosystems), according to the manufacturer's instructions. Libraries were pooled and sequenced 75 bp paired‐end on the NextSeq 500 (Illumina) using NextSeq High Output Kit v2 reagents (Illumina).

### 4.7 ATAC‐seq data analyses

We obtained ATAC‐seq data from spleen, PBL and sorted T cells from spleen of B6 and NZO mouse strains at age 3, 12, and 18 months. Paired‐end ATAC‐seq reads were quality trimmed using *Trimmomatic* (Bolger et al., 2014) and trimmed reads were aligned to mouse genome (mm10) using *BWA* (Li, 2013). After preprocessing and quality filtering, peaks were called on alignments with *MACS2* using the BAMPE option (Zhang et al., 2008). The consensus peakset for PCA and PVCA plots were generated gathering all peaks from all tissue/cell types, whereas for differential accessibility analyses the samples of the same tissue were merged to generate one consensus peak set by using R package *DiffBind* (Stark & Brown, 2011). Peaks only present in at least two samples were included in the analysis. Raw read counts were normalized using the *cpm* function via the log option turned on from edgeR package (Robinson et al., 2010).

### 4.8 Statistical methods

Principal variance component analysis (PVCA) was used in order to determine the sources of variability in flow cytometry data (Li, Bushel, et al., 2009), which combines the strengths of principal component analysis (PCA) and variance component analyses (VCA). So, using PVCA the proportions variances were attributed to each factor. To compare the normalized gene expressions and peak counts across different tissues and cell types, Wilcoxon rank sum test was used.

### 4.9 Differential analyses

To identify differentially expressed genes and differentially accessible peaks between age groups, we used the R package *edgeR* (Robinson et al., 2010) was used. It fits a generalized linear model (GLM) that includes age as a continuous independent variable and read counts from either ATAC‐seq or RNA‐seq as dependent variables to test for the effect of age on read counts. We stratified data by tissue and strain and fit GLM within strata. In addition to the age, we included sex as a covariate, which did not yield any statistically significant results. *p*‐Values for the age effect were adjusted using the Benjamini–Hochberg procedure, and genes or peaks with FDR‐adjusted *p*‐Value < 0.05 were considered differential.

To uncover genes that are the most significantly and robustly associated with aging, we calculated a similarity score based on the magnitude of association of genes (MAG (Luo et al., 2021)) to a phenotype. The MAG score was calculated by computing the geometric mean of the inverse of ranks of a gene for the two strains. The genes were then ranked based on the summation of the MAG score for each gene across the four studied cells/tissues. This ranked gene list was provided as input to the gene set enrichment analysis program using AP‐1 genes and age‐associated T‐cell markers as gene sets.

### 4.10 Enrichment analyses

Immune modules were obtained from human PBMCs (Chaussabel et al., 2008). Human and mouse orthologs were identified using R package *biomatrix* (Smedley et al., 2009). Consensus peaks were annotated using *HOMER* (Heinz et al., 2010) and gene‐based analyses were restricted to promoter peaks annotated to the nearest transcription start sites (TSS) of expressed genes. Hypergeometric p‐value is calculated for each module of the inflammation genesets. Then, *p*‐values are adjusted for multiple hypothesis testing using Benjamini–Hochberg correction. For all analyses, modules that have FDR‐adjusted *p*‐value <0.05 considered as enriched.

### 4.11 Single‐cell data analyses

We have downloaded 10× single‐cell RNAseq spleen data of Tabula Muris Senis (Consortium, 2020) from USCS browser (https://cells.ucsc.edu/?ds=tabula‐muris‐senis+droplet+spleen) and transferred it into R environment (v4.0.5). Next, we selected 3‐ and 18‐months samples (2 samples per age point) and ran the standard Seurat (v4.0.2) pipeline with the default parameters (log normalization, 10 PCs, and UMAP for dimensionality reduction). Then, we removed the cell types which had less than 100 cells in total along with the doublet cluster, remaining cells from four major cell types (NK, Macrophage, B, and T cells) were used in all single‐cell related analyses. We detected the cluster cell types based on their respective marker genes here: B cell (*Blnk*, *Cd79a*, *Cd79b*), T cell (*Cd3d*, *Cd3e*, *Cd3g*), NK (*Ncr1*, *Gzma*), and Macrophage (*Itgax*). We used MAST to compare young (3 mo) and old (18 mo) mice spleen samples *at single‐cell level*.

### 4.12 Footprinting analyses

ATAC‐seq data from spleen and PBL were scanned for TF footprints using the PIQ algorithm (Sherwood et al., 2014). This method integrates genome‐wide TF motifs (i.e., position weight matrices) with chromatin accessibility profiles to generate a list of potential TF binding sites that are bound by a TF. The method also produces a quality score for each footprint (positive predictive value). Only the TF footprints with positive predictive values >0.9 are used in downstream enrichment analyses.

Before footprint calling, we merged samples of the same sex, strain, tissue and age group to increase read depth, which improved the quality and detection power of PIQ. In addition to that we used *SAMtools* (Li, Handsaker, et al., 2009) to randomly downsample aligned reads from each merged data set to 50 M reads to minimize the impact of the high correlation between library depth and footprint positive predictive values. We used a set of motifs available in the JASPAR 2016 database (*n* = 454 for human and *n* = 189 for mouse) (Mathelier et al., 2016). Finally, footprint calls were further filtered to include in analyses only those associated with TFs that are expressed in the PBL or spleen. For each cell type, we applied *BiFET* (Youn et al., 2019) to identify TFs whose footprints are significantly more detected in opening/closing peaks compared to background peaks (peaks whose chromatin accessibility do not change with age). In each tissue, we selected TFs whose *BiFET* q‐values are <0.05 for at least two samples. We followed the same protocols for our previously published human chromatin accessibility data (Márquez et al., 2020) and merged PIQ calls of young (<40 years) and older individuals (>65 years). For AP‐1 complex related TF analyses, we selected each subunit from JASPAR annotated files. Then, the locations of these TFs were annotated to their closest TSS using *ChIPseeker* package (Yu et al., 2015) (v1.27.3) to uncover the most effected sites. Finally, we calculated enrichment scores of these sites using hypergeometric *p*‐value test and our immune signature gene sets. We used HINT‐ATAC (Li et al., 2019) which is part of the Regulatory Genomics Toolbox (RGT). The consensus peak files for young (3 months) and old mice (18 months) along with respective merged bam files were provided as input to the HINT program. To find TFs associated with a particular cellular condition, we checked for motifs overlapping with predicted footprints to find motif predicted binding sites (MPBS) with JASPAR motifs. Finally, HINT was used to generate average ATAC‐seq profiles around binding sites of a TF.

### 4.13 Cell stimulation and immunoblotting experiments

Mice spleens were removed aseptically and transferred to a Petri dish where they were minced and filtered through a 40‐μm nylon cell strainer containing DMEM medium. The cellular suspension was centrifuged to yield a pellet and then depleted of erythrocytes by resuspending the cells in pre‐chilled red blood cell lysis solution (Sigma). The spleen cells were washed twice in DMEM medium containing 25 mM HEPES, 1 mM l‐glutamine, 1% penicillin/streptomycin, and 10% heat‐inactivated FCS. Viability of the spleen cells was >90 %. Five million spleen cells per condition were suspended at 1 × 10^6^/mL in 10‐mL Petri dishes pre‐coated with 100 μg anti‐CD3/CD28 or in culture medium supplemented or not with either LPS (1 μg/mL) or Poly(I:C) (both high and low molecular weight, 10 μg/mL each) (Invivogen). After 2 h, cells were lysed, and the nuclear extracts were isolated using Nuclear Extraction Kit (Abcam) according to the manufacturer's instructions. Protein content of the nuclear extracts was quantified by Pierce™ Coomassie (Bradford) Protein Assay Kit (Thermo‐Fisher) and the activation of the JUN transcription factor was assayed by c‐Jun Transcription Factor Assay Kit (Abcam) according to the manufacturer's instruction. Secretion of IL‐6 was quantified from the supernatant of 10 million spleen cells stimulated as above, after 24 h using Mouse IL‐6 ELISA kit (Abcam).

The capillary immunoblotting analysis was performed, using Wes (ProteinSimple, Santa Clara, CA, USA), according to the ProteinSimple user manual. The lysates of the primary male mice splenocytes were mixed with a master mix (ProteinSimple) to a final concentration of 1 × sample buffer, 1 × fluorescent molecular weight marker, and 40 mM dithiothreitol and then heated at 95°C for 5 min. The samples, blocking reagents, primary antibodies, HRP‐conjugated secondary antibodies, chemiluminescent substrate (ProteinSimple), and separation and stacking matrices were also dispensed to the designated wells in a 25‐well plate. After plate loading, the separation electrophoresis and immunodetection steps took place in the capillary system and were fully automated. A capillary immunoblotting analysis was carried out at room temperature, and the instrument's default settings were used. Capillaries were first filled with a separation matrix followed by a stacking matrix, with about 40 nL of the sample used for loading. During electrophoresis, the proteins were separated by molecular weight through the stacking and separation matrices at 250 volts for 40–50 min and then immobilized on the capillary wall, using proprietary photo‐activated capture chemistry. The matrices were then washed out. The capillaries were next incubated with a blocking reagent for 15 min, and the target proteins were immunoprobed with primary antibodies followed by HRP‐conjugated secondary antibodies The antibodies of GAPDH (sc‐25778, 1:200, Santa Cruz Biotechnology), Lamin B1 (12586S, 1:100; Cell Signaling Technology), and c‐Jun (9165S, 1:50; Cell Signaling Technology) were diluted in an antibody diluent (ProteinSimple).

## 5 AUTHOR CONTRIBUTIONS

DU designed the study with help from JB, MH, and RK. OK, NK, and AY analyzed the data. RK, MH, and RM collected samples and generated data. MH, RM, and CC conducted functional experiments. DU, OK, and MH wrote the paper. SS developed the R Shiny app. All authors read and edited the manuscript.

## 7 CONFLICT OF INTEREST STATEMENT

The authors declare no competing financial interests. While this work was performed and the manuscript was being prepared, J.B. served on the Board of Directors (BOD) for Neovacs; served on the Scientific Advisory Board (SAB) for Georgiamune LLC; BOD member and a stockholder for Ascend Biopharmaceuticals; Scientific Advisory Board (SAB) member and a stockholder for Cue Biopharma; and a stockholder for Sanofi. JAX (J.B.) and Sanofi entered into a collaborative research agreement to work on a long‐read sequencing project that is not related (ended in Jul 2021). Since Aug 2021, J.B. joined Immunai in New York as Chief Scientific Officer (CSO) and continued a limited affiliation with JAX until the end of Feb 2022.

8

## Supporting information


Figure S1

Figure S2

Figure S3

Figure S4

Figure S5

Figure S6

Figure S7

Figure S8

Table S1

Table S2

Table S3

Table S4

Table S5

Table S6

Table S7

Table S8

Table S9

Table S10

Table S11

Table S12

Table S13

Table S14

Table S15

Table S16

Table S17

Table S18

Table S19

## Data Availability

Raw fastq and processed read count files for all samples are deposited to GEO accession code GSE159798. Code availability: The code for figures is deposited at https://github.com/UcarLab/mice‐aging‐project

## References

[acel13792-bib-0001] Ahmed, S. M. U. , Luo, L. , Namani, A. , Wang, X. J. , & Tang, X. (2017). Nrf2 signaling pathway: Pivotal roles in inflammation. Biochimica et Biophysica Acta (BBA) ‐ Molecular Basis of Disease, 1863(2), 585–597. 27825853 10.1016/j.bbadis.2016.11.005

[acel13792-bib-0002] Angel, P. , & Karin, M. (1991). The role of Jun, Fos and the AP‐1 complex in cell‐proliferation and transformation. Biochimica et Biophysica Acta (BBA)–Reviews on Cancer, 1072(2–3), 129–157. 1751545 10.1016/0304-419x(91)90011-9

[acel13792-bib-0003] Austad, S. N. , & Fischer, K. E. (2016). Sex differences in lifespan. Cell Metabolism, 23(6), 1022–1033. 27304504 10.1016/j.cmet.2016.05.019 PMC4932837

[acel13792-bib-0004] Benayoun, B. A. , Pollina, E. A. , Singh, P. P. , Mahmoudi, S. , Harel, I. , Casey, K. M. , Dulken, B. W. , Kundaje, A. , & Brunet, A. (2019). Remodeling of epigenome and transcriptome landscapes with aging in mice reveals widespread induction of inflammatory responses. Genome Research, 29(4), 697–709. 30858345 10.1101/gr.240093.118 PMC6442391

[acel13792-bib-0005] Bolger, A. M. , Lohse, M. , & Usadel, B. (2014). Trimmomatic: a flexible trimmer for Illumina sequence data. Bioinformatics, 30(15), 2114–2120. 24695404 10.1093/bioinformatics/btu170 PMC4103590

[acel13792-bib-0006] Boraschi, D. , Aguado, M. T. , Dutel, C. , Goronzy, J. , Louis, J. , Grubeck‐Loebenstein, B. , Rappuoli, R. , & Del Giudice, G. (2013). The gracefully aging immune system. Science Translational Medicine, 5, 185. 10.1126/scitranslmed.3005624 23677590

[acel13792-bib-0007] Buchmann, J. , Meyer, C. , Neschen, S. , Augustin, R. , Schmolz, K. , Kluge, R. , Al‐Hasani, H. , Jurgens, H. , Eulenberg, K. , Wehr, R. , Dohrmann, C. , Joost, H. G. , & Schurmann, A. (2007). Ablation of the cholesterol transporter adenosine triphosphate‐binding cassette transporter G1 reduces adipose cell size and protects against diet‐induced obesity. Endocrinology, 148(4), 1561–1573. 17194745 10.1210/en.2006-1244

[acel13792-bib-0008] Buenrostro, J. D. , Wu, B. , Chang, H. Y. , & Greenleaf, W. J. (2015). ATAC‐seq: a method for assaying chromatin accessibility genome‐wide. Current Protocols in Molecular Biology, 109(1), 21. 10.1002/0471142727.mb2129s109 PMC4374986 25559105

[acel13792-bib-0009] Chaussabel, D. , Quinn, C. , Shen, J. , Patel, P. , Glaser, C. , Baldwin, N. , Stichweh, D. , Blankenship, D. , Li, L. , & Munagala, I. (2008). A modular analysis framework for blood genomics studies: application to systemic lupus erythematosus. Immunity, 29(1), 150–164. 18631455 10.1016/j.immuni.2008.05.012 PMC2727981

[acel13792-bib-0010] Chen, J. , Flurkey, K. , & Harrison, D. E. (2002). A reduced peripheral blood CD4^+^ lymphocyte proportion is a consistent ageing phenotype. Mechanisms of Ageing and Development, 123(2‐3), 145–153. 11718808 10.1016/s0047-6374(01)00347-5

[acel13792-bib-0011] Consortium, T. M. (2020). A single cell transcriptomic atlas characterizes aging tissues in the mouse. Nature, 583(7817), 590. 32669714 10.1038/s41586-020-2496-1 PMC8240505

[acel13792-bib-0012] Foletta, V. C. , Segal, D. H. , & Cohen, D. R. (1998). Transcriptional regulation in the immune system: all roads lead to AP‐1. Journal of Leukocyte Biology, 63(2), 139–152. 9468273 10.1002/jlb.63.2.139

[acel13792-bib-0013] Folgueras, A. R. , Freitas‐Rodríguez, S. , Velasco, G. , & López‐Otín, C. (2018). Mouse models to disentangle the hallmarks of human aging. Circulation Research, 123(7), 905–924. 30355076 10.1161/CIRCRESAHA.118.312204

[acel13792-bib-0014] Franceschi, C. , & Campisi, J. (2014). Chronic inflammation (inflammaging) and its potential contribution to age‐associated diseases. The Journals of Gerontology. Series A, Biological Sciences and Medical Sciences, 69(Suppl 1), S4–S9. 24833586 10.1093/gerona/glu057

[acel13792-bib-0015] Fujioka, S. , Niu, J. , Schmidt, C. , Sclabas, G. M. , Peng, B. , Uwagawa, T. , Li, Z. , Evans, D. B. , Abbruzzese, J. L. , & Chiao, P. J. (2004). NF‐κB and AP‐1 connection: mechanism of NF‐κB‐dependent regulation of AP‐1 activity. Molecular and Cellular Biology, 24(17), 7806–7819. 15314185 10.1128/MCB.24.17.7806-7819.2004 PMC507000

[acel13792-bib-0016] Furman, D. (2015). Sexual dimorphism in immunity: improving our understanding of vaccine immune responses in men. Expert Review of Vaccines, 14(3), 461–471. 25278153 10.1586/14760584.2015.966694

[acel13792-bib-0017] Furman, D. , Campisi, J. , Verdin, E. , Carrera‐Bastos, P. , Targ, S. , Franceschi, C. , Ferrucci, L. , Gilroy, D. W. , Fasano, A. , & Miller, G. W. (2019). Chronic inflammation in the etiology of disease across the life span. Nature Medicine, 25(12), 1822–1832. 10.1038/s41591-019-0675-0 PMC7147972 31806905

[acel13792-bib-0018] Furman, D. , Hejblum, B. P. , Simon, N. , Jojic, V. , Dekker, C. L. , Thiébaut, R. , Tibshirani, R. J. , & Davis, M. M. (2014). Systems analysis of sex differences reveals an immunosuppressive role for testosterone in the response to influenza vaccination. Proceedings of the National Academy of Sciences, 111(2), 869–874. 10.1073/pnas.1321060111 PMC3896147 24367114

[acel13792-bib-0019] Goronzy, J. J. , Hu, B. , Kim, C. , Jadhav, R. R. , & Weyand, C. M. (2018). Epigenetics of T cell aging. Journal of Leukocyte Biology, 104(4), 691–699. 29947427 10.1002/JLB.1RI0418-160R PMC6162101

[acel13792-bib-0020] Gusmao, E. G. , Allhoff, M. , Zenke, M. , & Costa, I. G. (2016). Analysis of computational footprinting methods for DNase sequencing experiments. Nature Methods, 13(4), 303–309. 26901649 10.1038/nmeth.3772

[acel13792-bib-0021] Gusmao, E. G. , Dieterich, C. , Zenke, M. , & Costa, I. G. (2014). Detection of active transcription factor binding sites with the combination of DNase hypersensitivity and histone modifications. Bioinformatics, 30(22), 3143–3151. 25086003 10.1093/bioinformatics/btu519

[acel13792-bib-0022] Heinz, S. , Benner, C. , Spann, N. , Bertolino, E. , Lin, Y. C. , Laslo, P. , Cheng, J. X. , Murre, C. , Singh, H. , & Glass, C. K. (2010). Simple combinations of lineage‐determining transcription factors prime cis‐regulatory elements required for macrophage and B cell identities. Molecular Cell, 38(4), 576–589. 20513432 10.1016/j.molcel.2010.05.004 PMC2898526

[acel13792-bib-0023] Hess, J. , Angel, P. , & Schorpp‐Kistner, M. (2004). AP‐1 subunits: quarrel and harmony among siblings. Journal of Cell Science, 117(25), 5965–5973. 15564374 10.1242/jcs.01589

[acel13792-bib-0024] Igel, M. , Becker, W. , Herberg, L. , & Joost, H. G. (1997). Hyperleptinemia, leptin resistance, and polymorphic leptin receptor in the New Zealand obese mouse. Endocrinology, 138(10), 4234–4239. 9322935 10.1210/endo.138.10.5428

[acel13792-bib-0025] Ji, Z. , He, L. , Regev, A. , & Struhl, K. (2019). Inflammatory regulatory network mediated by the joint action of NF‐kB, STAT3, and AP‐1 factors is involved in many human cancers. Proceedings of the National Academy of Sciences, 116(19), 9453–9462. 10.1073/pnas.1821068116 PMC6511065 30910960

[acel13792-bib-0026] Karin, M. , Liu, Z.‐G. , & Zandi, E. (1997). AP‐1 function and regulation. Current Opinion in Cell Biology, 9(2), 240–246. 9069263 10.1016/s0955-0674(97)80068-3

[acel13792-bib-0027] Khan, A. , Fornes, O. , Stigliani, A. , Gheorghe, M. , Castro‐Mondragon, J. A. , Van Der Lee, R. , Bessy, A. , Cheneby, J. , Kulkarni, S. R. , & Tan, G. (2018). JASPAR 2018: update of the open‐access database of transcription factor binding profiles and its web framework. Nucleic Acids Research, 46(D1), D260–D266. 29140473 10.1093/nar/gkx1126 PMC5753243

[acel13792-bib-0028] Langmead, B. , & Salzberg, S. L. (2012). Fast gapped‐read alignment with Bowtie 2. Nature Methods, 9(4), 357–359. 22388286 10.1038/nmeth.1923 PMC3322381

[acel13792-bib-0029] Lawlor, N. , Nehar‐Belaid, D. , Grassmann, J. D. S. , Stoeckius, M. , Smibert, P. , Stitzel, M. L. , Pascual, V. , Banchereau, J. , Williams, A. , & Ucar, D. (2021). Single cell analysis of blood mononuclear cells stimulated through either LPS or anti‐CD3 and anti‐CD28. Frontiers in Immunology, 12, 691. 10.3389/fimmu.2021.636720 PMC8010670 33815388

[acel13792-bib-0030] Li, B. , & Dewey, C. N. (2011). RSEM: accurate transcript quantification from RNA‐Seq data with or without a reference genome. BMC Bioinformatics, 12(1), 1–16. 21816040 10.1186/1471-2105-12-323 PMC3163565

[acel13792-bib-0031] Li, H. (2013). Aligning sequence reads, clone sequences and assembly contigs with BWA‐MEM. arXiv Preprint, arXiv:13033997.

[acel13792-bib-0032] Li, H. , Handsaker, B. , Wysoker, A. , Fennell, T. , Ruan, J. , Homer, N. , Marth, G. , Abecasis, G. , & Durbin, R. (2009). The sequence alignment/map format and SAMtools. Bioinformatics, 25(16), 2078–2079. 19505943 10.1093/bioinformatics/btp352 PMC2723002

[acel13792-bib-0033] Li, J. , Bushel, P. R. , Chu, T. M. , & Wolfinger, R. D. (2009). Principal variance components analysis: estimating batch effects in microarray gene expression data. In A. Scherer (Ed.), Batch Effects and Noise in Microarray Experiments: Sources and Solutions (pp. 141–154). Wiley.

[acel13792-bib-0034] Li, Z. , Schulz, M. H. , Look, T. , Begemann, M. , Zenke, M. , & Costa, I. G. (2019). Identification of transcription factor binding sites using ATAC‐seq. Genome Biology, 20(1), 1–21. 30808370 10.1186/s13059-019-1642-2 PMC6391789

[acel13792-bib-0035] Luo, D. , Zhang, C. , Fu, L. , Zhang, Y. , & Hu, Y.‐Q. (2021). A novel similarity score based on gene ranks to reveal genetic relationships among diseases. PeerJ, 9, e10576. 33505797 10.7717/peerj.10576 PMC7796663

[acel13792-bib-0036] Maggio, M. , Guralnik, J. M. , Longo, D. L. , & Ferrucci, L. (2006). Interleukin‐6 in aging and chronic disease: a magnificent pathway. The Journals of Gerontology Series A: Biological Sciences and Medical Sciences, 61(6), 575–584. 16799139 10.1093/gerona/61.6.575 PMC2645627

[acel13792-bib-0037] Márquez, E. J. , Chung, C.‐H. , Marches, R. , Rossi, R. J. , Nehar‐Belaid, D. , Eroglu, A. , Mellert, D. J. , Kuchel, G. A. , Banchereau, J. , & Ucar, D. (2020). Sexual‐dimorphism in human immune system aging. Nature Communications, 11(1), 1–17. 10.1038/s41467-020-14396-9 PMC7005316 32029736

[acel13792-bib-0038] Mathelier, A. , Fornes, O. , Arenillas, D. J. , Chen, C.‐Y. , Denay, G. , Lee, J. , Shi, W. , Shyr, C. , Tan, G. , & Worsley‐Hunt, R. (2016). JASPAR 2016: a major expansion and update of the open‐access database of transcription factor binding profiles. Nucleic Acids Research, 44(D1), D110–D115. 26531826 10.1093/nar/gkv1176 PMC4702842

[acel13792-bib-0039] Matsumoto, M. , & Seya, T. (2008). TLR3: interferon induction by double‐stranded RNA including poly(I:C). Advanced Drug Delivery Reviews, 60(7), 805–812. 18262679 10.1016/j.addr.2007.11.005

[acel13792-bib-0040] Melez, K. A. , Harrison, L. C. , Gilliam, J. N. , & Steinberg, A. D. (1980). Diabetes is associated with autoimmunity in the New Zealand obese (NZO) mouse. Diabetes, 29(10), 835–840. 7002665 10.2337/diacare.20.10.835

[acel13792-bib-0041] Mogilenko, D. A. , Shpynov, O. , Andhey, P. S. , Arthur, L. , Swain, A. , Esaulova, E. , Brioschi, S. , Shchukina, I. , Kerndl, M. , & Bambouskova, M. (2021). Comprehensive profiling of an aging immune system reveals clonal GZMK^+^ CD8^+^ T cells as conserved hallmark of inflammaging. Immunity, 54(1), 99–115. 33271118 10.1016/j.immuni.2020.11.005

[acel13792-bib-0042] Moskowitz, D. M. , Zhang, D. W. , Hu, B. , Le Saux, S. , Yanes, R. E. , Ye, Z. , Buenrostro, J. D. , Weyand, C. M. , Greenleaf, W. J. , & Goronzy, J. J. (2017). Epigenomics of human CD8 T cell differentiation and aging. Science Immunology, 2(8), eaag0192. 28439570 10.1126/sciimmunol.aag0192 PMC5399889

[acel13792-bib-0043] Nikolich‐Žugich, J. (2008). Ageing and life‐long maintenance of T‐cell subsets in the face of latent persistent infections. Nature Reviews. Immunology, 8(7), 512–522. 10.1038/nri2318 PMC5573867 18469829

[acel13792-bib-0044] Pan, H. J. , Agate, D. S. , King, B. L. , Wu, M. K. , Roderick, S. L. , Leiter, E. H. , & Cohen, D. E. (2006). A polymorphism in New Zealand inbred mouse strains that inactivates phosphatidylcholine transfer protein. FEBS Letters, 580(25), 5953–5958. 17046758 10.1016/j.febslet.2006.09.066 PMC1693963

[acel13792-bib-0045] Pauken, K. E. , & Wherry, E. J. (2015). SnapShot: T cell exhaustion. Cell, 163(4), 1038. 26544946 10.1016/j.cell.2015.10.054

[acel13792-bib-0046] Peters, M. J. , Joehanes, R. , Pilling, L. C. , Schurmann, C. , Conneely, K. N. , Powell, J. , Reinmaa, E. , Sutphin, G. L. , Zhernakova, A. , & Schramm, K. (2015). The transcriptional landscape of age in human peripheral blood. Nature Communications, 6(1), 1–14. 10.1038/ncomms9570 PMC4639797 26490707

[acel13792-bib-0047] Pinchuk, L. M. , & Filipov, N. M. (2008). Differential effects of age on circulating and splenic leukocyte populations in C57BL/6 and BALB/c male mice. Immunity & Ageing, 5(1), 1–12. 18267021 10.1186/1742-4933-5-1 PMC2268915

[acel13792-bib-0048] Renoux, F. , Stellato, M. , Haftmann, C. , Vogetseder, A. , Huang, R. , Subramaniam, A. , Becker, M. O. , Blyszczuk, P. , Becher, B. , & Distler, J. H. (2020). The AP1 transcription factor Fosl2 promotes systemic autoimmunity and inflammation by repressing Treg development. Cell Reports, 31(13), 107826. 32610127 10.1016/j.celrep.2020.107826

[acel13792-bib-0049] Robinson, M. D. , McCarthy, D. J. , & Smyth, G. K. (2010). edgeR: a Bioconductor package for differential expression analysis of digital gene expression data. Bioinformatics, 26(1), 139–140. 19910308 10.1093/bioinformatics/btp616 PMC2796818

[acel13792-bib-0050] SanMiguel, J. M. , Young, K. , & Trowbridge, J. J. (2020). Hand in hand: intrinsic and extrinsic drivers of aging and clonal hematopoiesis. Experimental Hematology, 91, 1–9. 32991978 10.1016/j.exphem.2020.09.197 PMC7736302

[acel13792-bib-0051] Shaulian, E. , & Karin, M. (2002). AP‐1 as a regulator of cell life and death. Nature Cell Biology, 4(5), E131–E136. 11988758 10.1038/ncb0502-e131

[acel13792-bib-0052] Sherwood, R. I. , Hashimoto, T. , O'donnell, C. W. , Lewis, S. , Barkal, A. A. , Van Hoff, J. P. , Karun, V. , Jaakkola, T. , & Gifford, D. K. (2014). Discovery of directional and nondirectional pioneer transcription factors by modeling DNase profile magnitude and shape. Nature Biotechnology, 32(2), 171–178. 10.1038/nbt.2798 PMC3951735 24441470

[acel13792-bib-0053] Smedley, D. , Haider, S. , Ballester, B. , Holland, R. , London, D. , Thorisson, G. , & Kasprzyk, A. (2009). BioMart–biological queries made easy. BMC Genomics, 10(1), 1–12. 19144180 10.1186/1471-2164-10-22 PMC2649164

[acel13792-bib-0054] Srivastava, R. M. , Marincola, F. M. , & Shanker, A. (2019). Lymphocyte functional crosstalk and regulation. Frontiers Media SA, 10, 2916. 10.3389/fimmu.2019.02916 PMC6914817 31921175

[acel13792-bib-0055] Stark, R. , & Brown, G. (2011). DiffBind: differential binding analysis of ChIP‐Seq peak data. R Package Version, 100.

[acel13792-bib-0056] Stowell, N. C. , Seideman, J. , Raymond, H. A. , Smalley, K. A. , Lamb, R. J. , Egenolf, D. D. , Bugelski, P. J. , Murray, L. A. , Marsters, P. A. , & Bunting, R. A. (2009). Long‐term activation of TLR3 by poly(I:C) induces inflammation and impairs lung function in mice. Respiratory Research, 10(1), 1–14. 19486528 10.1186/1465-9921-10-43 PMC2694181

[acel13792-bib-0057] Tan, J. T. , Ernst, B. , Kieper, W. C. , LeRoy, E. , Sprent, J. , & Surh, C. D. (2002). Interleukin (IL)‐15 and IL‐7 jointly regulate homeostatic proliferation of memory phenotype CD8^+^ cells but are not required for memory phenotype CD4^+^ cells. The Journal of Experimental Medicine, 195(12), 1523–1532. 12070280 10.1084/jem.20020066 PMC2193564

[acel13792-bib-0058] Tan, R. S. , Ho, B. , Leung, B. P. , & Ding, J. L. (2014). TLR cross‐talk confers specificity to innate immunity. International Reviews of Immunology, 33(6), 443–453. 24911430 10.3109/08830185.2014.921164 PMC4266099

[acel13792-bib-0059] Ucar, D. , Marquez, E. J. , Chung, C. H. , Marches, R. , Rossi, R. J. , Uyar, A. , Wu, T. C. , George, J. , Stitzel, M. L. , Palucka, A. K. , Kuchel, G. A. , & Banchereau, J. (2017). The chromatin accessibility signature of human immune aging stems from CD8(+) T cells. The Journal of Experimental Medicine, 214, 3123–3144. 28904110 10.1084/jem.20170416 PMC5626401

[acel13792-bib-0060] Vogel, H. , Scherneck, S. , Kanzleiter, T. , Benz, V. , Kluge, R. , Stadion, M. , Kryvych, S. , Bluher, M. , Kloting, N. , Joost, H. G. , & Schurmann, A. (2012). Loss of function of Ifi202b by a microdeletion on chromosome 1 of C57BL/6J mice suppresses 11beta‐hydroxysteroid dehydrogenase type 1 expression and development of obesity. Human Molecular Genetics, 21(17), 3845–3857. 22692684 10.1093/hmg/dds213

[acel13792-bib-0061] Wagner, E. F. (2001). AP‐1–Introductory remarks. Oncogene, 20(19), 2334–2335. 11402330 10.1038/sj.onc.1204416

[acel13792-bib-0062] Weyand, C. M. , & Goronzy, J. J. (2016). Aging of the immune system. Mechanisms and therapeutic targets. Annals of the American Thoracic Society, 13, S422–S428. 28005419 10.1513/AnnalsATS.201602-095AW PMC5291468

[acel13792-bib-0063] Wimmers, F. , Donato, M. , Kuo, A. , Ashuach, T. , Gupta, S. , Li, C. , Dvorak, M. , Foecke, M. H. , Chang, S. E. , & Hagan, T. (2021). The single‐cell epigenomic and transcriptional landscape of immunity to influenza vaccination. Cell, 184, 3915–3935. 34174187 10.1016/j.cell.2021.05.039 PMC8316438

[acel13792-bib-0064] Wu, Z. , Nicoll, M. , & Ingham, R. J. (2021). AP‐1 family transcription factors: A diverse family of proteins that regulate varied cellular activities in classical hodgkin lymphoma and ALK^+^ ALCL. Experimental Hematology & Oncology, 10(1), 1–12. 33413671 10.1186/s40164-020-00197-9 PMC7792353

[acel13792-bib-0065] Youn, A. , Marquez, E. J. , Lawlor, N. , Stitzel, M. L. , & Ucar, D. (2019). BiFET: sequencing bias‐free transcription factor footprint enrichment test. Nucleic Acids Research, 47(2), e11. 30428075 10.1093/nar/gky1117 PMC6344870

[acel13792-bib-0066] Yu, G. , Wang, L.‐G. , & He, Q.‐Y. (2015). ChIPseeker: an R/Bioconductor package for ChIP peak annotation, comparison and visualization. Bioinformatics, 31(14), 2382–2383. 25765347 10.1093/bioinformatics/btv145

[acel13792-bib-0067] Yuan, R. , Tsaih, S. W. , Petkova, S. B. , De Evsikova, C. M. , Xing, S. , Marion, M. A. , Bogue, M. A. , Mills, K. D. , Peters, L. L. , & Bult, C. J. (2009). Aging in inbred strains of mice: study design and interim report on median lifespans and circulating IGF1 levels. Aging Cell, 8(3), 277–287. 19627267 10.1111/j.1474-9726.2009.00478.x PMC2768517

[acel13792-bib-0068] Zhang, H. , Weyand, C. M. , & Goronzy, J. J. (2021). Hallmarks of the aging T‐cell system. The FEBS Journal, 288(24), 7123–7142. 33590946 10.1111/febs.15770 PMC8364928

[acel13792-bib-0069] Zhang, Y. , Liu, T. , Meyer, C. A. , Eeckhoute, J. , Johnson, D. S. , Bernstein, B. E. , Nusbaum, C. , Myers, R. M. , Brown, M. , & Li, W. (2008). Model‐based analysis of ChIP‐Seq (MACS). Genome Biology, 9(9), 1–9. 10.1186/gb-2008-9-9-r137 PMC2592715 18798982

