# Supplementary material for: Transcriptional activation of Jun and Fos members of the AP‐1 complex is a conserved signature of immune aging that contributes to inflammaging
Source: Aging Cell. 2023 Feb 24;22(4):e13792. doi: 10.1111/acel.13792 (PMC10086525; doi:10.1111/acel.13792)

Figure S1

A Number of samples per age

		B6			NZO		
	months	3	12	18	3	12	18
Flow Cytometry	*PBL*	5	0	6	6	0	6
	spleen	11	11	6	6	11	6
ATACseq	*PBL*	5	0	5	6	0	5
	spleen	4	1	5	5	6	6
	CD8+ memory	5	0	5	6	0	6
	CD8+ naive	3	0	5	6	0	6
RNAseq	*PBL*	5	0	6	6	0	6
	spleen	5	6	6	6	6	6
	CD8+ memory	5	0	6	6	0	6
	CD8+ naive	5	0	5	6	0	6

E Percentage of variances explained by meta data with interactions (PVCA)

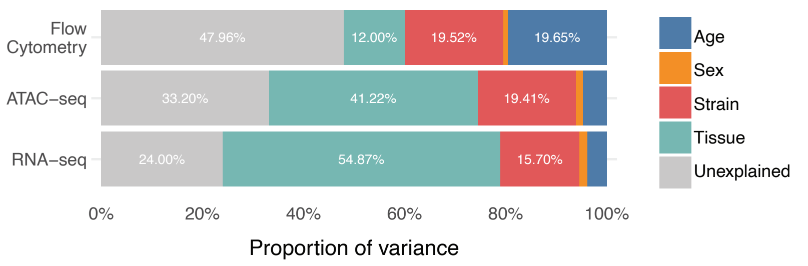

B PCA plots for flow cytometry

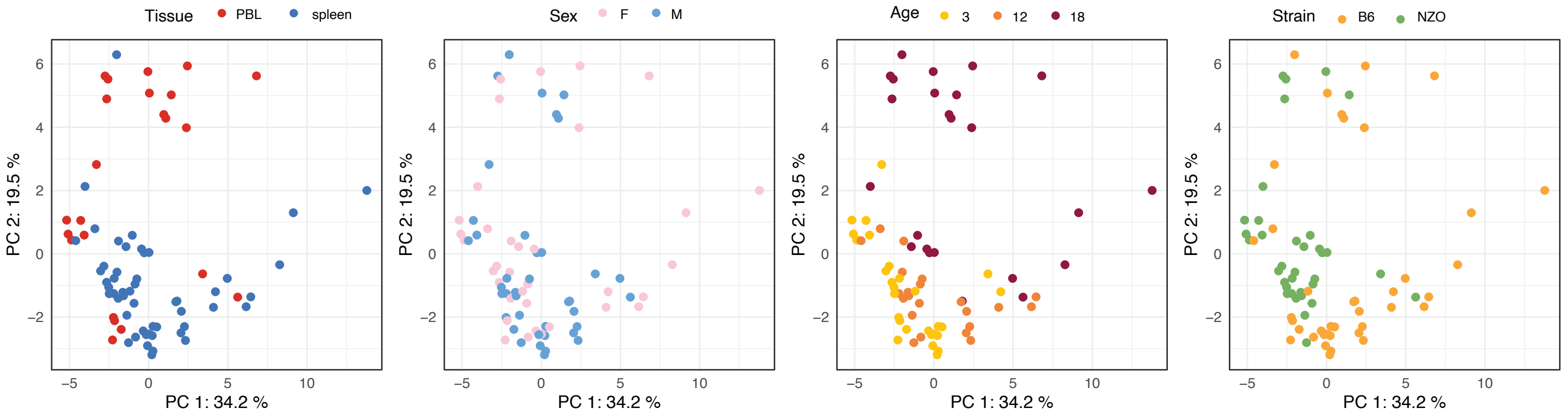

C PCA plots for ATAC-seq data

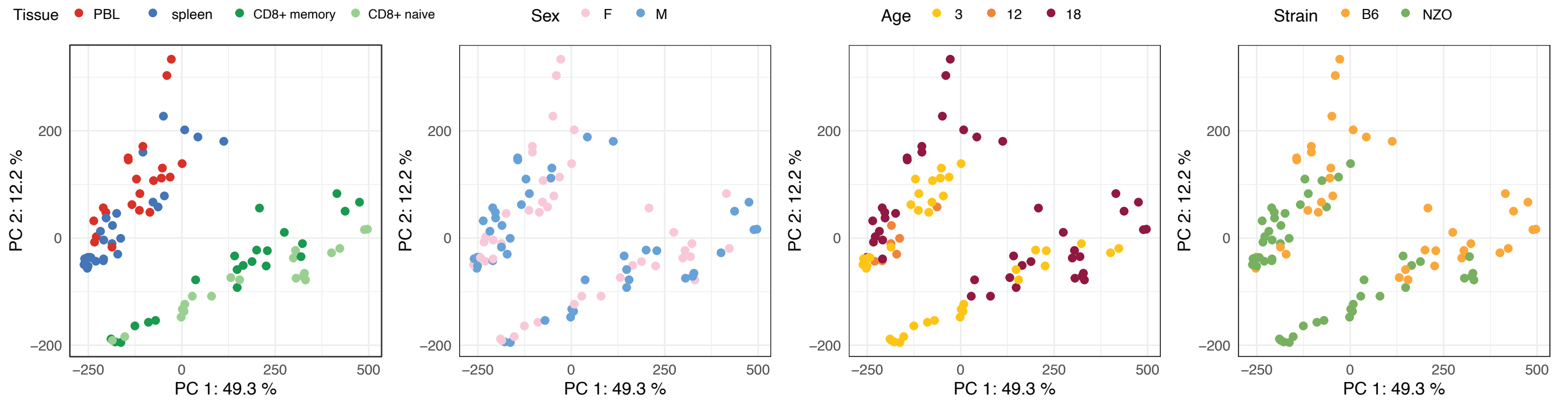

D PCA plots for RNA-seq data

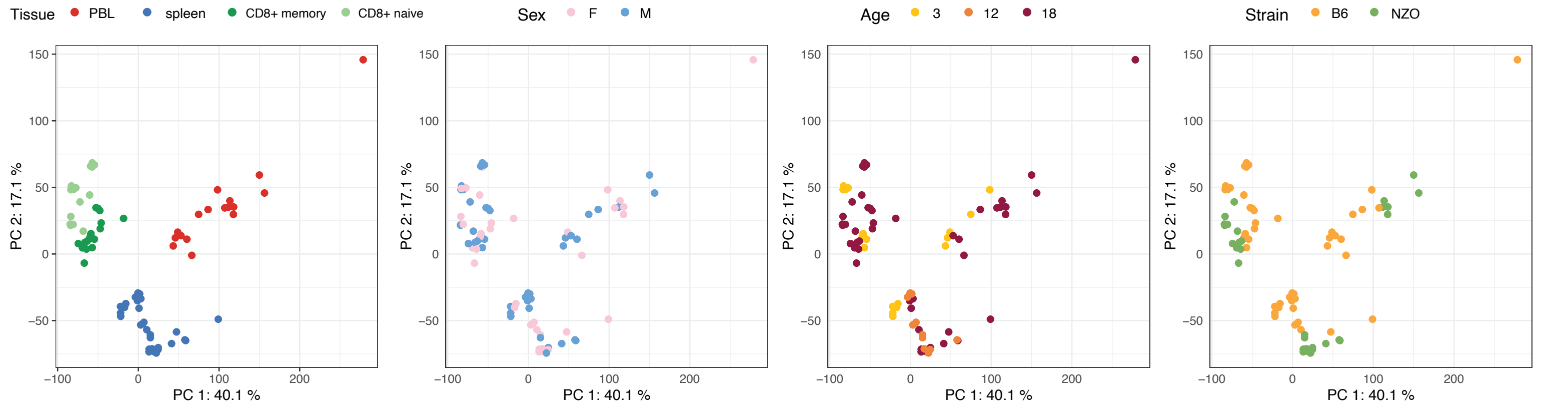

Supplement: Supplementary file 1 — Figure S1 [file ACEL-22-e13792-s011.pdf]
