# Supplementary figures and images for: Transcriptional activation of Jun and Fos members of the AP‐1 complex is a conserved signature of immune aging that contributes to inflammaging
Source: Aging Cell. 2023 Feb 24;22(4):e13792. doi: 10.1111/acel.13792 (PMC10086525; doi:10.1111/acel.13792)

## Figure S2

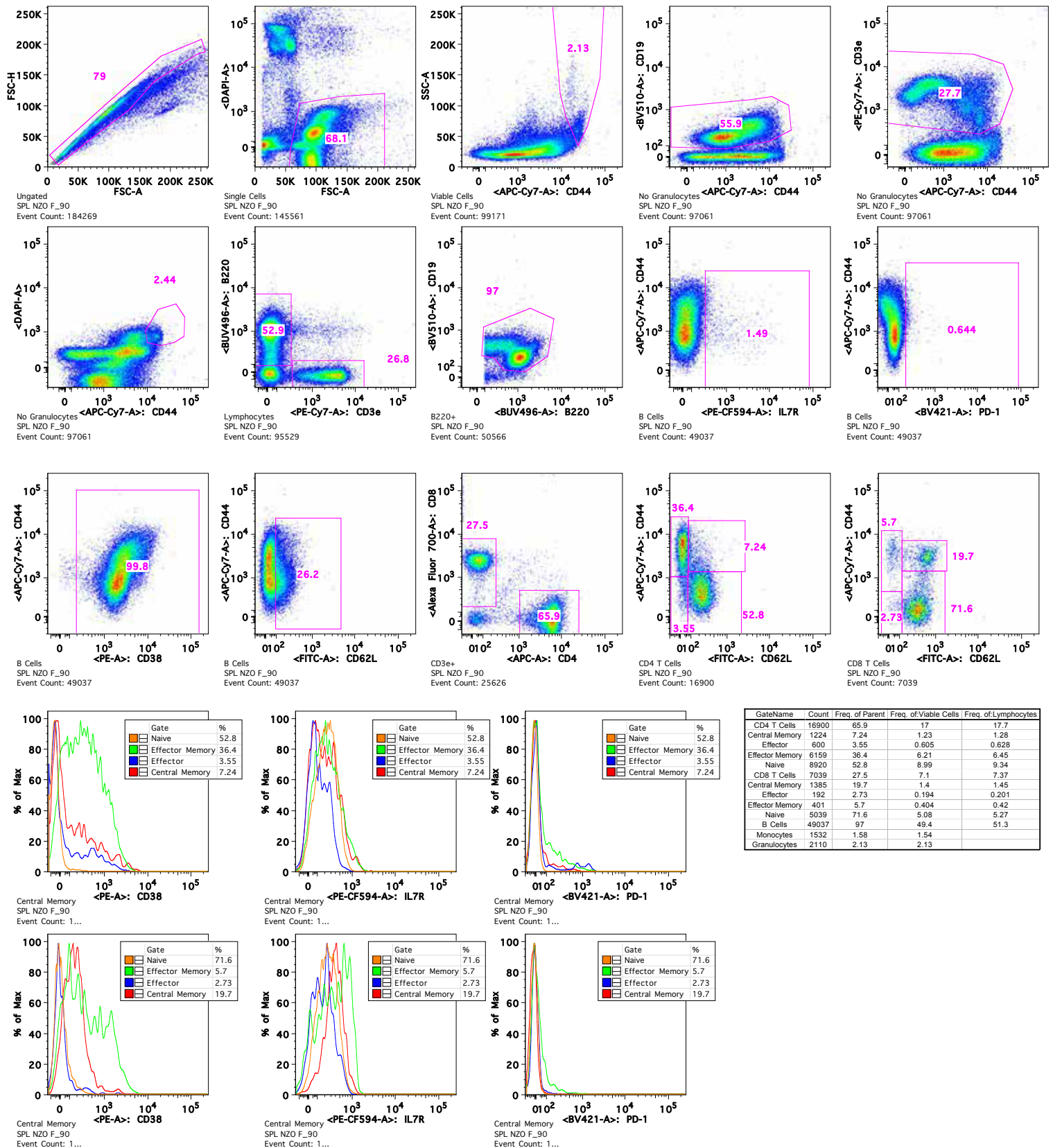

Supplement: Supplementary file 2 — Figure S2 [file ACEL-22-e13792-s013.pdf]

Figure S6

Memory

Naive

PBL

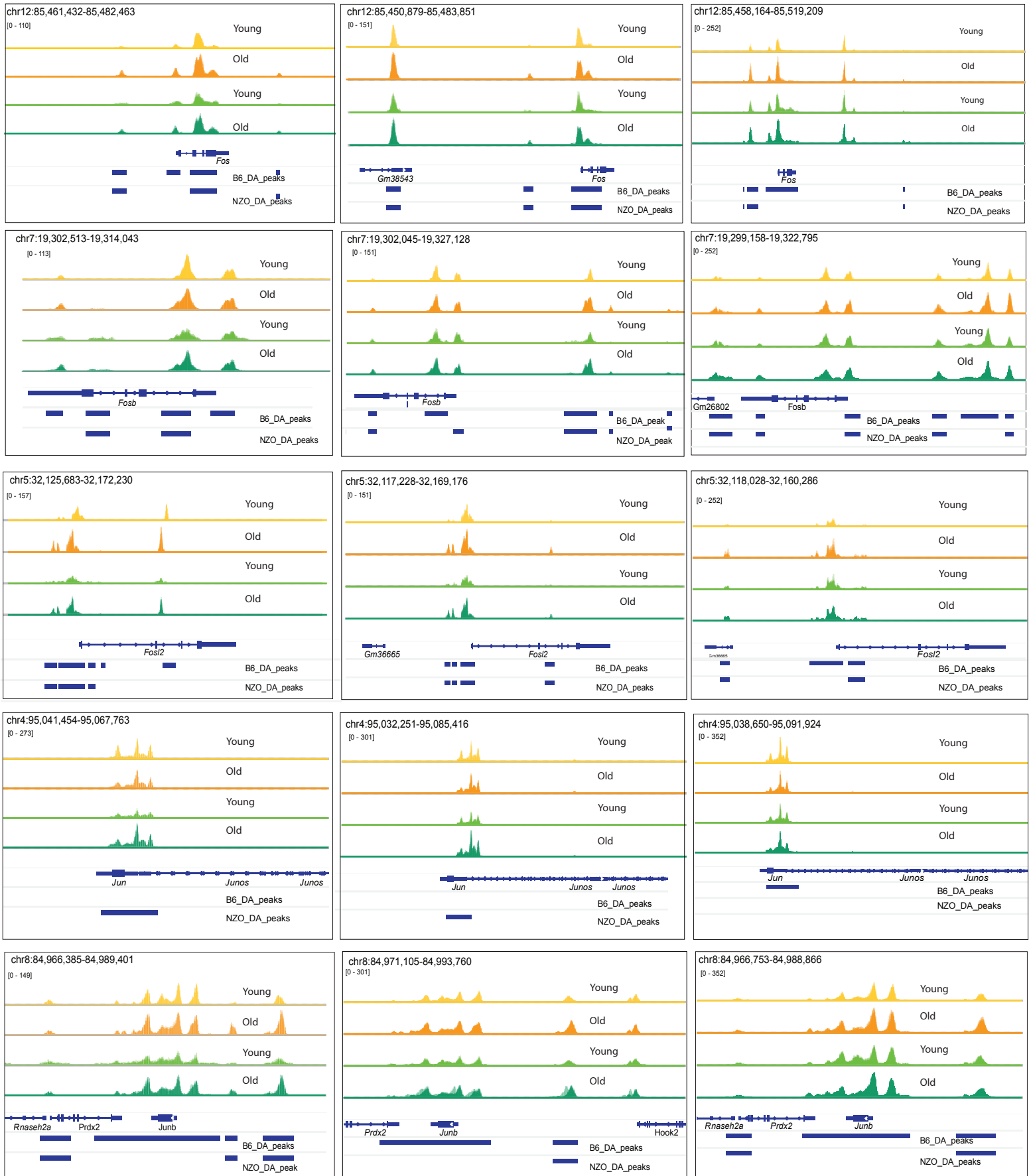

} B6
 

} NZO

Supplement: Supplementary file 6 — Figure S6 [file ACEL-22-e13792-s006.pdf]

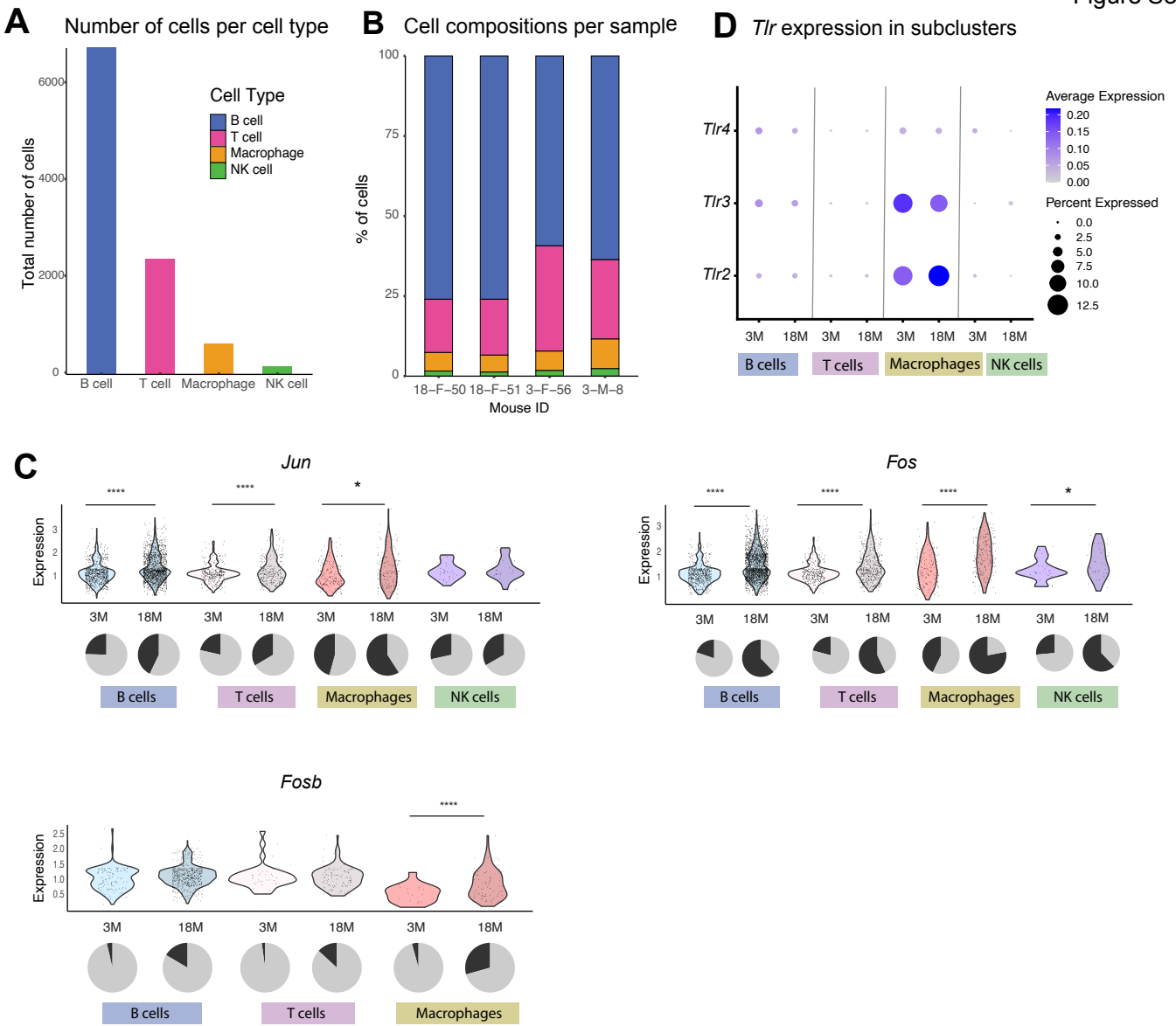

Supplement: Supplementary file 8 — Figure S8 [file ACEL-22-e13792-s004.pdf]
