# Supplementary material for: Transcriptional activation of Jun and Fos members of the AP‐1 complex is a conserved signature of immune aging that contributes to inflammaging
Source: Aging Cell. 2023 Feb 24;22(4):e13792. doi: 10.1111/acel.13792 (PMC10086525; doi:10.1111/acel.13792)

Figure S3

A All cell-compositonal changes in mice

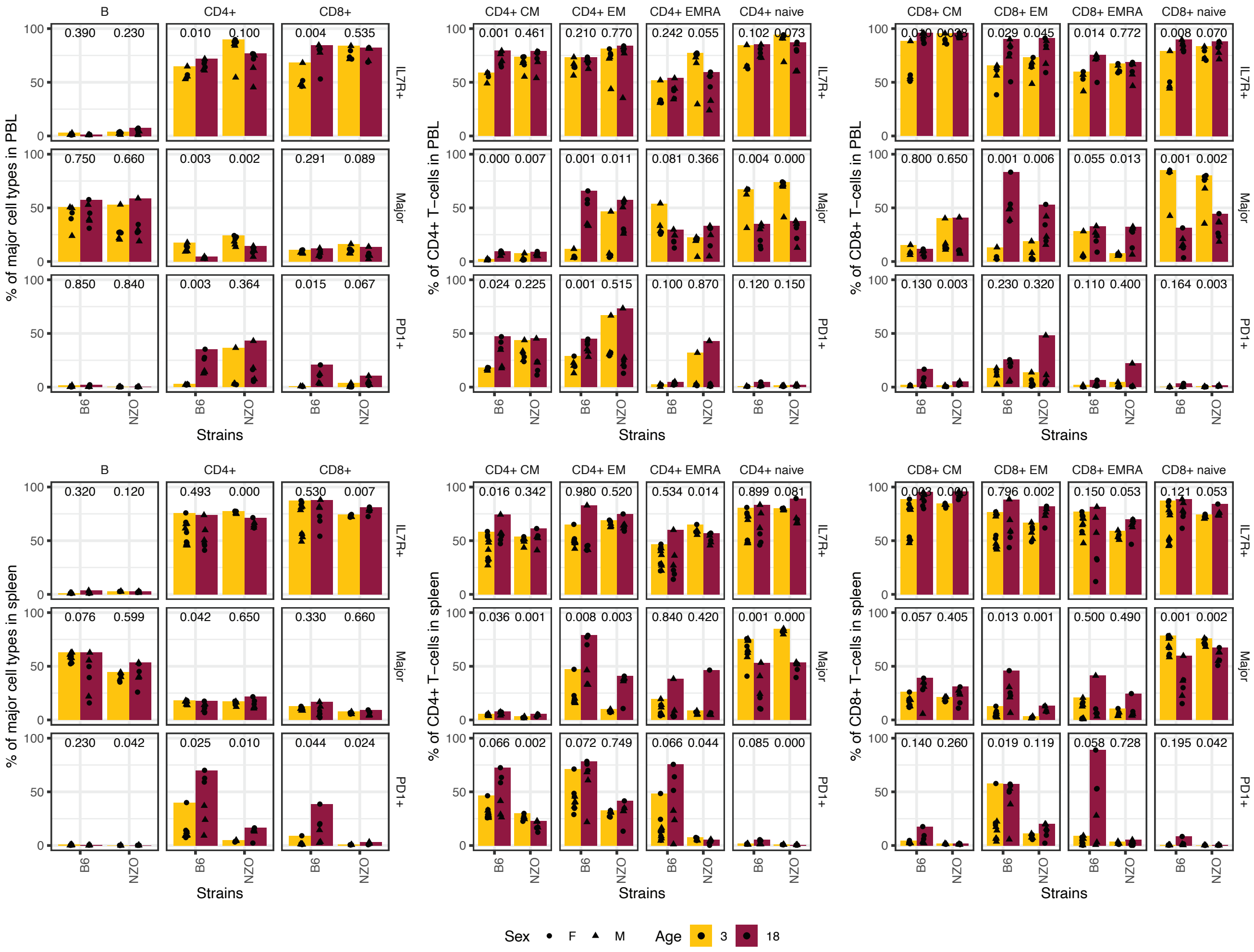

B Age and cell composition associations for IL7R/PD1 populations (mice)

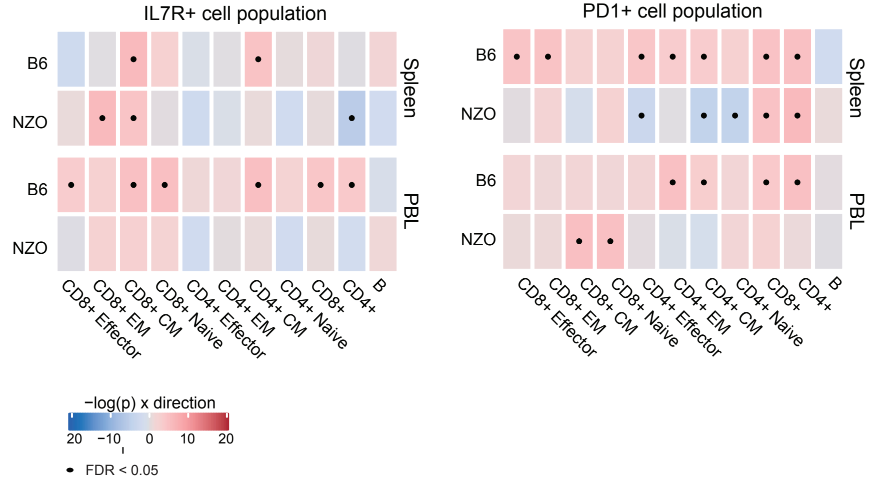

Supplement: Supplementary file 3 — Figure S3 [file ACEL-22-e13792-s023.pdf]
