# Supplementary material for: Transcriptional activation of Jun and Fos members of the AP‐1 complex is a conserved signature of immune aging that contributes to inflammaging
Source: Aging Cell. 2023 Feb 24;22(4):e13792. doi: 10.1111/acel.13792 (PMC10086525; doi:10.1111/acel.13792)
Supplement: Supplementary file 4 — Figure S4 [file ACEL-22-e13792-s021.pdf]

Figure S4

## A Functional enrichments of age-related changes

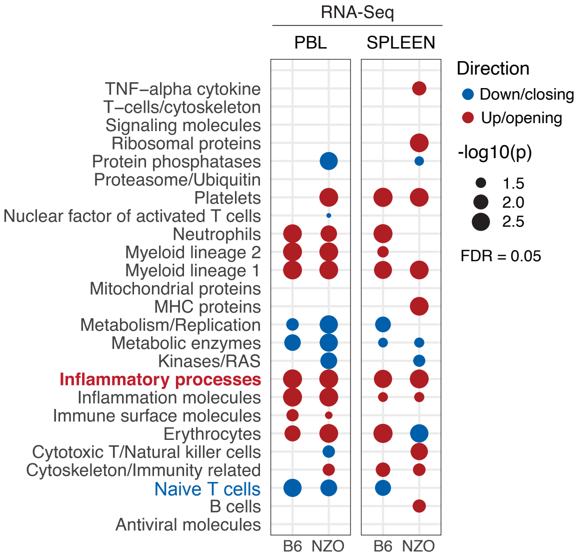


## B GZMK+ T cell markers

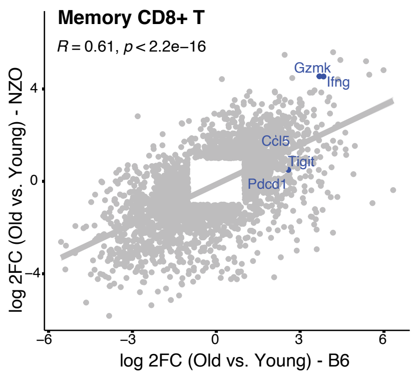


## C NES (normalized enrichment score) from GSEA

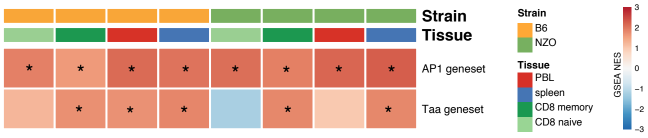


## D Age-related changes of T-cells in AP1 subfamilies

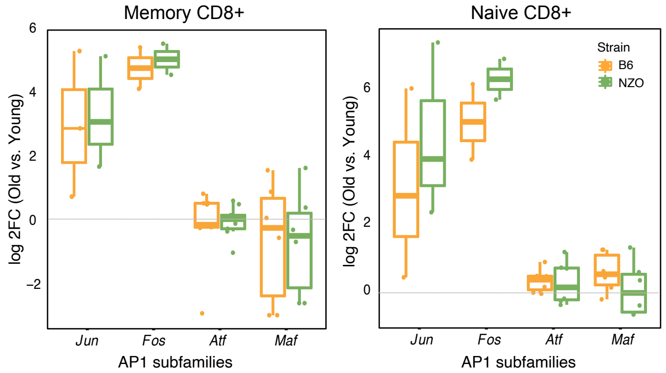


## E DE genes (Upregulated)

| | B6 ONLY | NZO ONLY | COMMON | pval |
|---|---|---|---|---|
| MEMORY | 538 | 426 | 407 | <2.2e-16 |
| NAIVE | 649 | 298 | 250 | <2.2e-16 |
| PBL | 386 | 255 | 210 | <2.2e-16 |
| SPLEEN | 715 | 641 | 407 | <2.2e-16 |

## F DE genes (Downregulated)

| | B6 ONLY | NZO ONLY | COMMON | pval |
|---|---|---|---|---|
| MEMORY | 3318 | 2172 | 4133 | <2.2e-16 |
| NAIVE | 4774 | 1670 | 2258 | <2.2e-16 |
| PBL | 3244 | 1728 | 2344 | <2.2e-16 |
| SPLEEN | 3649 | 3582 | 3058 | <2.2e-16 |
