# Supplementary material for: Transcriptional activation of Jun and Fos members of the AP‐1 complex is a conserved signature of immune aging that contributes to inflammaging
Source: Aging Cell. 2023 Feb 24;22(4):e13792. doi: 10.1111/acel.13792 (PMC10086525; doi:10.1111/acel.13792)
Supplement: Supplementary file 5 — Figure S5 [file ACEL-22-e13792-s008.pdf]

Figure S5

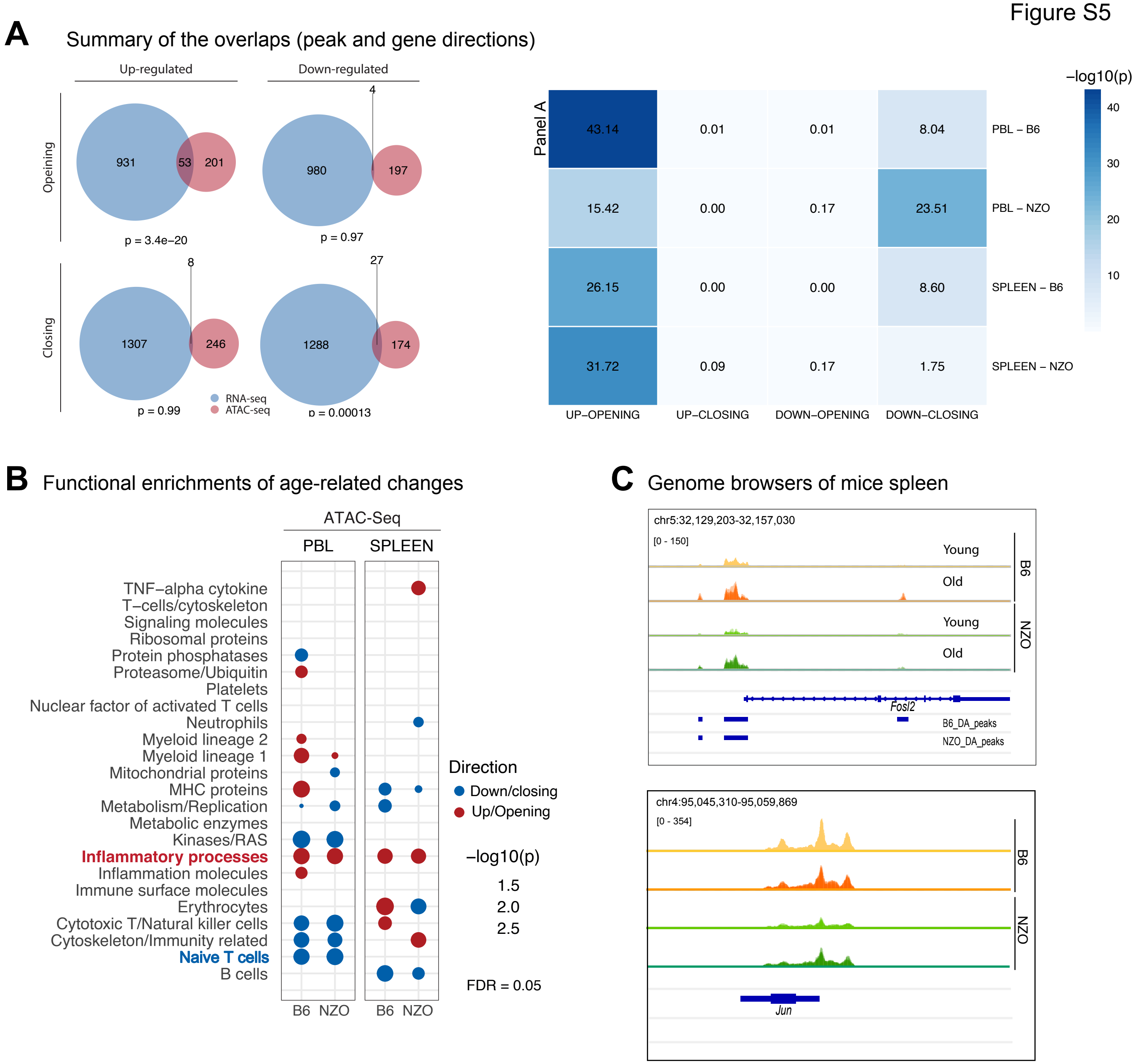
A
Summary of the overlaps (peak and gene directions)
Up-regulated
Down-regulated
Opeining
Closing
931
53
201
p = 3.4e−20
4
980
197
p = 0.97
8
1307
246
p = 0.99
27
1288
174
p = 0.00013
RNA-seq
ATAC-seq
Panel A
−log10(p)
40
30
20
10
43.14
0.01
0.01
8.04
PBL – B6
15.42
0.00
0.17
23.51
PBL – NZO
26.15
0.00
0.00
8.60
SPLEEN – B6
31.72
0.09
0.17
1.75
SPLEEN – NZO
UP−OPENING
UP−CLOSING
DOWN−OPENING
DOWN−CLOSING
B
Functional enrichments of age-related changes
ATAC-Seq
PBL
SPLEEN
TNF−alpha cytokine
T−cells/cytoskeleton
Signaling molecules
Ribosomal proteins
Protein phosphatases
Proteasome/Ubiquitin
Platelets
Nuclear factor of activated T cells
Neutrophils
Myeloid lineage 2
Myeloid lineage 1
Mitochondrial proteins
MHC proteins
Metabolism/Replication
Metabolic enzymes
Kinases/RAS
Inflammatory processes
Inflammation molecules
Immune surface molecules
Erythrocytes
Cytotoxic T/Natural killer cells
Cytoskeleton/Immunity related
Naive T cells
B cells
B6
NZO
B6
NZO
Direction
Down/closing
Up/Opening
−log10(p)
1.5
2.0
2.5
FDR = 0.05
C
Genome browsers of mice spleen
chr5:32,129,203-32,157,030
[0 - 150]
Young
Old
Young
Old
B6
NZO
Fosl2
B6_DA_peaks
NZO_DA_peaks
chr4:95,045,310-95,059,869
[0 - 354]
B6
NZO
Jun
