# Supplementary material for: Transcriptional activation of Jun and Fos members of the AP‐1 complex is a conserved signature of immune aging that contributes to inflammaging
Source: Aging Cell. 2023 Feb 24;22(4):e13792. doi: 10.1111/acel.13792 (PMC10086525; doi:10.1111/acel.13792)
Supplement: Supplementary file 7 — Figure S7 [file ACEL-22-e13792-s002.pdf]

Figure S7

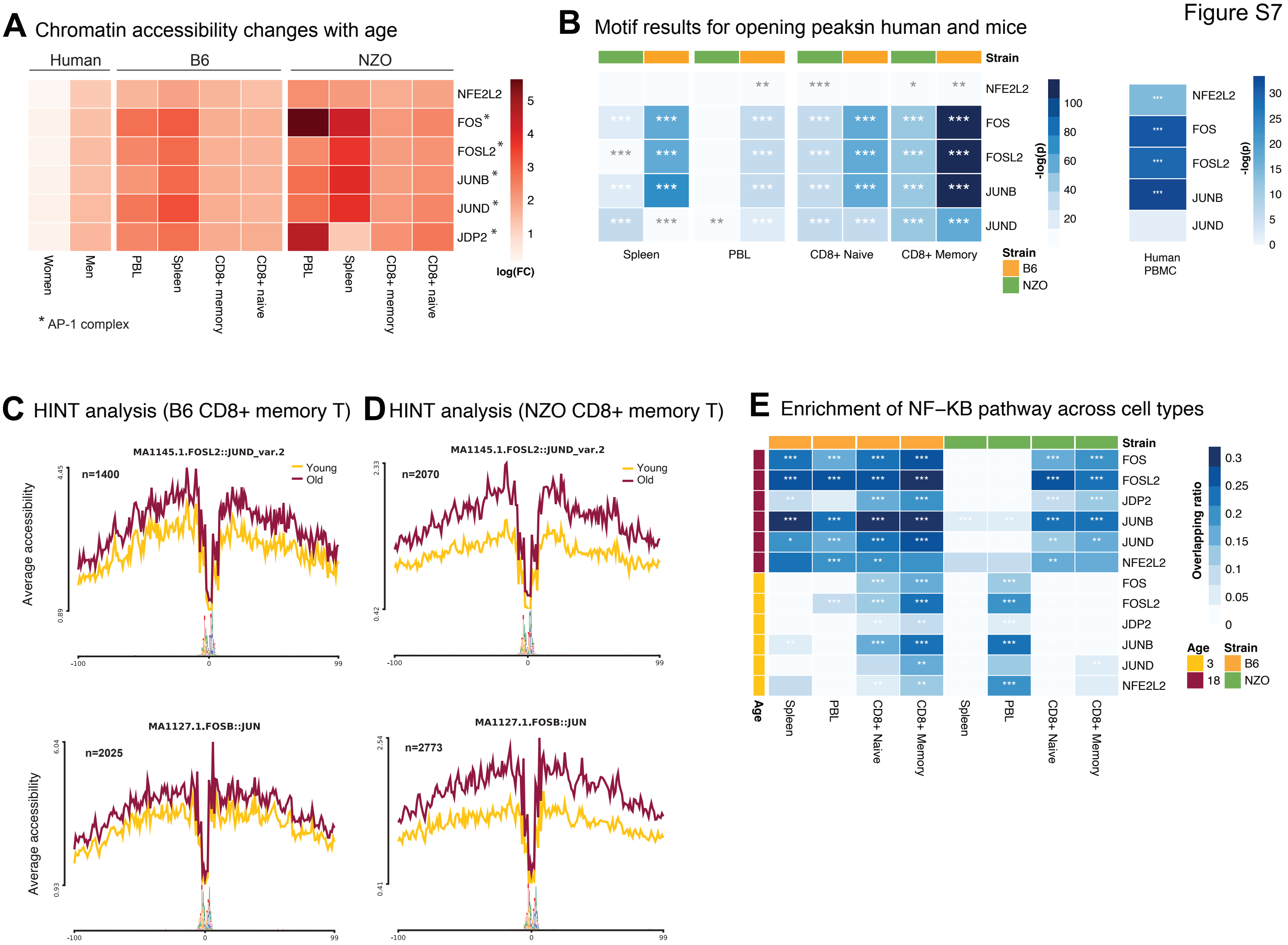

A Chromatin accessibility changes with age
Human
B6
NZO
Women
Men
PBL
Spleen
CD8+ memory
CD8+ naive
NFE2L2
FOS *
FOSL2 *
JUNB *
JUND *
JDP2 *
log(FC)
* AP-1 complex
B Motif results for opening peaksin human and mice
Strain
Spleen
PBL
CD8+ Naive
CD8+ Memory
-log(p)
Strain
B6
NZO
Human PBMC
C HINT analysis (B6 CD8+ memory T)
D HINT analysis (NZO CD8+ memory T)
MA1145.1.FOSL2::JUND_var.2
MA1127.1.FOSB::JUN
n=1400
n=2070
n=2025
n=2773
Young
Old
Average accessibility
E Enrichment of NF−KB pathway across cell types
Strain
Overlapping ratio
Age
3
18
B6
NZO
Spleen
PBL
CD8+ Naive
CD8+ Memory
